# Prevalence of Hypertension in Low- and Middle-Income Countries: A Systematic Review and Meta-Analysis

**DOI:** 10.1097/MD.0000000000001959

**Published:** 2015-12-18

**Authors:** Ahmed M. Sarki, Chidozie U. Nduka, Saverio Stranges, Ngianga-Bakwin Kandala, Olalekan A. Uthman

**Affiliations:** From the Division of Health Sciences, University of Warwick Medical School, Coventry, UK (AMS, CUN); Family and Youth Health Initiative (FAYOHI), Nigeria (AMS); Department of Population Health, Luxembourg Institute of Health, Luxembourg (SS, N-BK); Warwick-Centre for Applied Health Research and Delivery (WCAHRD), Division of health Sciences, University of Warwick Medical School, Coventry, UK (OAU); and Centre for Applied Health Research and Delivery (CAHRD), Liverpool School of Tropical Medicine, International Health Group, Liverpool, UK (OAU); Department of Mathematics and Information sciences, Faculty of Engineering and Environment, Northumbria University, Newcastle upon Tyne, UK (N-BK).

## Abstract

We aimed to obtain overall and regional estimates of hypertension prevalence, and to examine the pattern of this disease condition across different socio-demographic characteristics in low-and middle-income countries.

We searched electronic databases from inception to August 2015. We included population-based studies that reported hypertension prevalence using the current definition of blood pressure ≥140/90 mm Hg or self-reported use of antihypertensive medication. We used random-effects meta-analyses to pool prevalence estimates of hypertension, overall, by World Bank region and country income group. Meta-regression analyses were performed to explore sources of heterogeneity across the included studies.

A total of 242 studies, comprising data on 1,494,609 adults from 45 countries, met our inclusion criteria. The overall prevalence of hypertension was 32.3% (95% confidence interval [CI] 29.4–35.3), with the Latin America and Caribbean region reporting the highest estimates (39.1%, 95% CI 33.1–45.2). Pooled prevalence estimate was also highest across upper middle income countries (37.8%, 95% CI 35.0–40.6) and lowest across low-income countries (23.1%, 95% CI 20.1–26.2). Prevalence estimates were significantly higher in the elderly (≥65 years) compared with younger adults (<65 years) overall and across the geographical regions; however, there was no significant sex-difference in hypertension prevalence (31.9% vs 30.8%, *P* = 0.6). Persons without formal education (49.0% vs 24.9%, *P* < 0.00001), overweight/obese (46.4% vs 26.3%, *P* < 0.00001), and urban settlers (32.7% vs 25.2%, *P* = 0.0005) were also more likely to be hypertensive, compared with those who were educated, normal weight, and rural settlers respectively.

This study provides contemporary and up-to-date estimates that reflect the significant burden of hypertension in low- and middle-income countries, as well as evidence that hypertension remains a major public health issue across the various socio-demographic subgroups. On average, about 1 in 3 adults in the developing world is hypertensive. The findings of this study will be useful for the design of hypertension screening and treatment programmes in low- and middle-income countries.

## Abstract 2

Supplemental Digital Content is available in the text

## INTRODUCTION

Hypertension drives the global burden of cardiovascular disease, being widely acknowledged as the most common cardiovascular disorder and number 1 risk factor for mortality.^1–3^ The occurrence of hypertension is increasing globally, with projections estimating a 30% increase in prevalence by the year 2025.^4^ However, owing to several factors such as the ongoing nutritional transition, increasing trends in sedentary lifestyle, and other modifiable risk factors, and inadequate health care systems, populations in low- and middle-income countries (LMICs) may bear a higher burden of the disease, compared with the global average. Projections estimate that three-quarters of the world's hypertensive population will reside in LMICs within the next decade.^4^ However, there is a dearth of evidence providing up-to-date estimates of the occurrence of hypertension and its determinants across the developing regions of the world. The existing systematic reviews have, hitherto, been country-specific,^5^ or focused on African populations.^6,7^ Therefore, we aimed to fill this gap in the evidence by providing overall and regional estimates of hypertension prevalence across LMICs and to examine the pattern of this disease across different socio-demographic characteristics.

## METHODS

### Protocol and Registration

The systematic review rational and methods were specified in advance and documented in a protocol, which was published in the PROSPERO register (CRD42013006162).^8^

### Eligibility Criteria

We included population-based studies that reported crude prevalence estimates of hypertension in LMICs (see Table 1 for full inclusion and exclusion criteria).

**TABLE 1:**
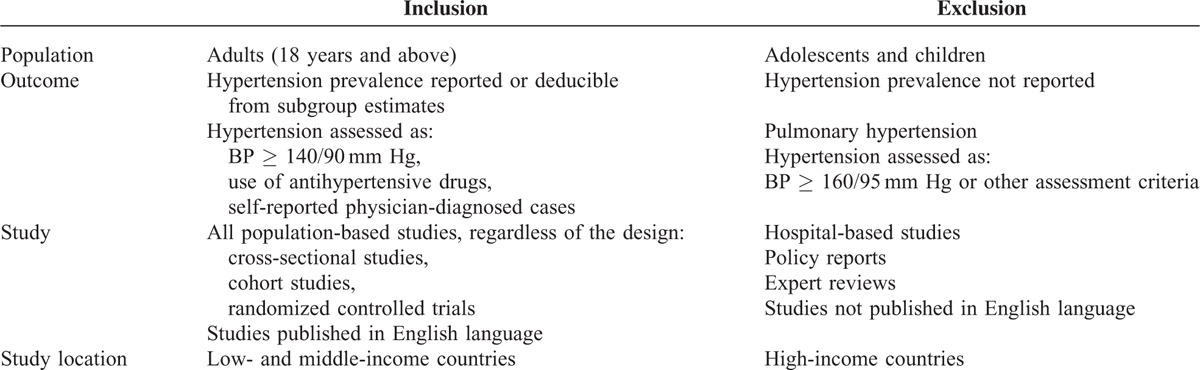
Eligibility criteria

### Information Sources and Search Strategy

We searched the following electronic databases from inception to August 2015: EMBASE, Ovid MEDLINE, and the WHO Global Cardiovascular Infobase for relevant articles. The search was conducted using the following medical subject heading (MeSH) terms and keywords: “hypertension” OR “blood pressure” OR “hypertens^∗^” AND “population-based” OR “etiology” OR “etiology” OR “prevalence” OR “epidemiolog^∗^” AND “low- and middle-income countries” OR “developing countries” (see Appendix 1 for the MEDLINE search strategy). We also scanned through cross-references of identified primary studies and review articles for eligible studies.

### Study Selection

Three reviewers (AMS, CN, and OU) independently evaluated the eligibility of the studies obtained from the literature searches. All articles yielded by the database search were initially screened by their titles and abstracts to obtain studies that met our inclusion criteria. In cases of discrepancies, agreement was reached by consensus and by discussion with a fourth reviewer (SS).

### Data Collection Process and Data Items

Three reviewers (AMS, CN, and OU) independently evaluated the methodological quality of each included study and extracted data using a piloted form; discrepancies were resolved by discussion with a fourth reviewer (SS). Data extracted included year of publication, country of origin, study design, sample size, sampling strategy, study period, setting (rural/urban), gender distribution, age group, mean age, body mass index (BMI) category, hypertension prevalence, diagnostic criteria for hypertension, confounders, smoking status, alcohol use, education and employment status. Countries were grouped by region and income according to World Bank^9^ development indicators. Age group, gender, BMI category, smoker status, alcohol use, and study setting were coded as dichotomous variables. We defined overweight/obesity as BMI ≥ 25 kg/m^2^. Total prevalence estimates of hypertension were calculated from studies providing only subgroup estimates. The total mean age in each study was also obtained from reports of this variable measure within subgroups.

### Risk of Bias in Included Studies

Methodological quality entailed assessing the risk of bias for each study using a domain-based tool adapted from the Newcastle-Ottawa Scale (see Appendix 2, http://links.lww.com/MD/A543).^10^ The risk of bias in each study was classified as low, moderate, high or unclear across the following domains: selection of participants (selection bias), sample size justification (selection bias), outcome measurement (detection bias), and confounding adjustment.

### Ethical Approval

Being a systematic review of published literature, no ethical approval was required for conducting this study. However, we ensured that all studies included in our review provided evidence of ethical approval and informed consent from all patients or respondents where required.

### Statistical Analysis

We stabilized the raw proportions of participants with hypertension from each study using the Freeman–Tukey variant of the arcsine square root transformed proportion^11^ suitable for pooling proportions (see Appendix 3, http://links.lww.com/MD/A543). In performing the meta-analyses, we used the DerSimonian–Laird random-effects model^12^ due to anticipated variations in study population, methodologies, and stage of epidemic transition. We assessed heterogeneity among studies by inspecting the forest plots and using the chi squared test for heterogeneity with a 10% level of statistical significance, and using the *I*^*2*^ statistic, where we interpreted a value of 50% as representing moderate heterogeneity.^13,14^ We assessed the potential for publication bias by evaluating funnel plot asymmetry using Egger's test for regression asymmetry.^15^ Where there was evidence of publication bias, we used the “trim and fill” analysis of Duval and Tweedie^16^ to examine the potential impact of missed or unpublished studies on the pooled estimates of hypertension prevalence.

The potential modifying effects of various study-level variables on the overall prevalence of hypertension were explored using subgroup and univariable random-effects meta-regression analyses: year of publication, age group, gender, education status, employment status, smoker status, alcohol use, overweight/obesity, country income groups,^9^ and study settings (urban, rural or mixed).

Hypertension prevalence estimates were reported with 95% confidence intervals (CIs). All *P* values were exact (except where *P* < 0.0001)^14^ and 2-tailed; *P* < 0.05 was considered statistically significant. Analyses were conducted using Stata version 12 for Windows (Stata Corp, College Station, TX) using the “metaprop” rountine.^17^ This systematic review was reported according to the Preferred Reporting Items for Systematic Reviews and Meta-analyses (PRISMA) guidelines (http://www.prisma-statement.org).^18^ The PRISMA checklist is provided in Appendix 4, http://links.lww.com/MD/A543.

## RESULTS

### Study Flow and Characteristics

**FIGURE 1:**
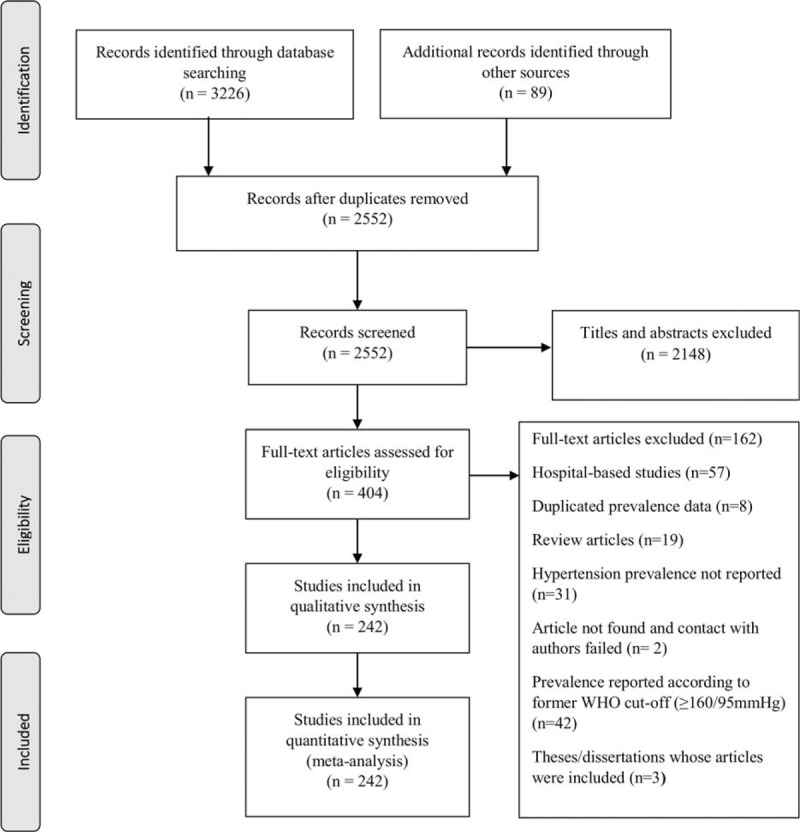
PRISMA flow diagram showing study selection.

Figure 1 shows the study selection flow. The literature search yielded 3315 articles. After an initial screening process, 404 articles were selected for critical reading. One hundred and sixty two (162) studies were excluded for not meeting the selection criteria, leaving 242 studies (from 239 publications),^19–254^ which we considered eligible for inclusion in this systematic review. Table 2 and eTables 1–6, http://links.lww.com/MD/A543 summarize the characteristics of the included studies by region.

**TABLE 2:**
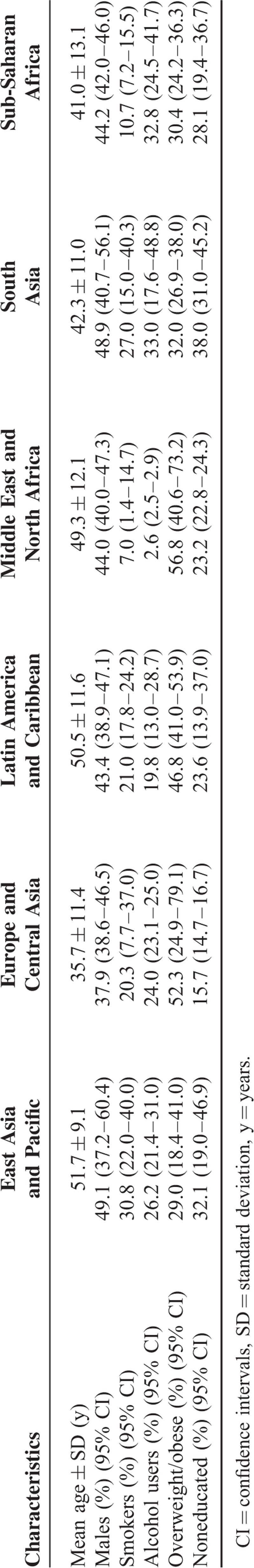
Characteristics of Included Studies by Regions

**FIGURE 2:**
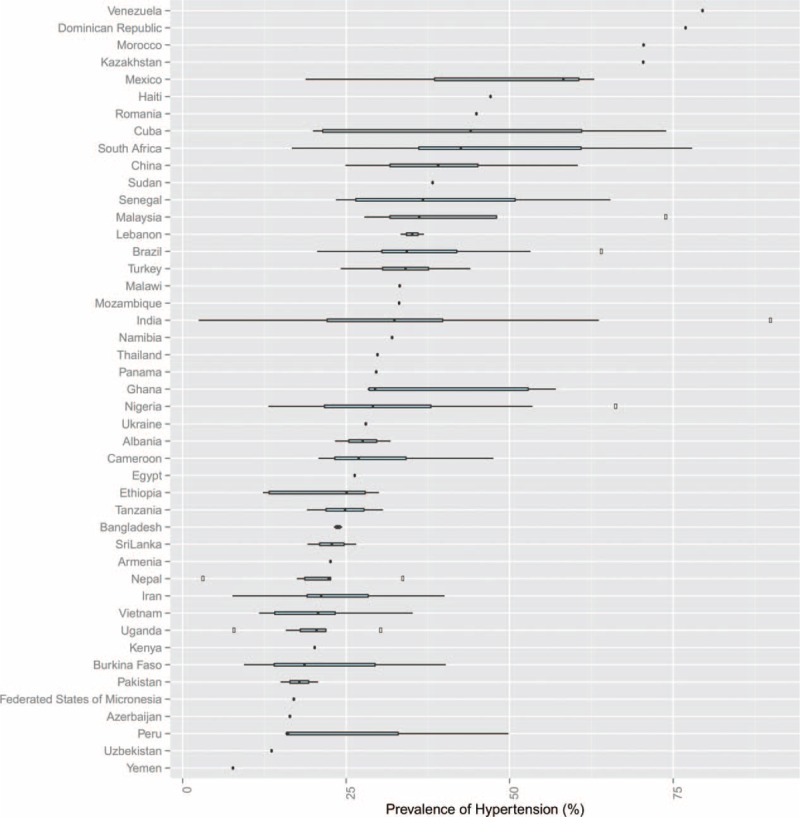
Prevalence estimates of hypertension by region, income group, and study setting.

Studies comprised data on 1,494,609 participants (52% females) from 45 countries (Table S7, http://links.lww.com/MD/A543). Most of the studies were conducted in India (n = 50 studies; 20.7%), Brazil (n = 26; 10.7%), Nigeria (n = 25; 10.3%), and China (n = 22; 9.1%). Although a third of the included studies (n = 74; 30.6%) originated from sub-Saharan African countries, all 6 regions, including East Asia and Pacific, Europe and Central Asia, Latin America and Caribbean, Middle East and North Africa, South Asia, and sub-Saharan Africa were represented in our review. Figure 2 showcases country-wide differences among the 45 countries included in this study.

All articles were population-based observational studies. Hypertension was diagnosed as blood pressure (BP) ≥140/90 mm Hg in all but 3 studies where assessment was based on self-reported physician diagnoses alone.^58,62,70^ The mean age overall was 45.9 ± 12.1 years with participants from Europe and Central Asia being the youngest (35.7 ± 11.4 years) and those from East Asia and Pacific being the oldest (51.7 ± 9.1 years). Participants in the Middle East and North Africa had the highest estimates of combined overweight/obesity (56.8% [95% CI 40.6–73.2]), whereas the lowest rates were found in the South Asia region (29.0% [95% CI 18.4–41.0]).

### Risk of Bias of Included Studies

Summary of risk of bias assessment for each study is shown in Appendix 5, http://links.lww.com/MD/A543. Overall, 111 studies (46%) were assessed as having low risk of selection bias within the sampling domain, having selected participants randomly. Based on sample size justification, the risk of selection bias was also assessed as low in 73 studies (30%). Detection bias was low in 193 studies (80%) reporting the use of a validated tool for measuring blood pressure. However, assessment of hypertension was not blind in any of the included studies, consequently resulting in high risk of detection bias within this domain in all 242 studies.

### Pooled Prevalence by Geographical Regions

The country-specific prevalence of hypertension is shown in Figure 2. All 242 studies reported the crude prevalence of hypertension and were included in meta-analysis. The reported hypertension ranged from 2.5% (95% CI 1.9–3.2)^107^ to 90% (95% CI 89.9–90.2)^29^ (both studies in India). The pooled hypertension prevalence for all studies yielded an estimate of 32.3% (95% CI 29.4–35.3). The *I*^*2*^ statistic was 99.9%, indicating substantial heterogeneity across the included studies. The contour-enhanced funnel plot for assessing publication bias is shown in eFigure 1, http://links.lww.com/MD/A543. The funnel plot appears symmetric and shows no evidence of publication bias.

**FIGURE 3:**
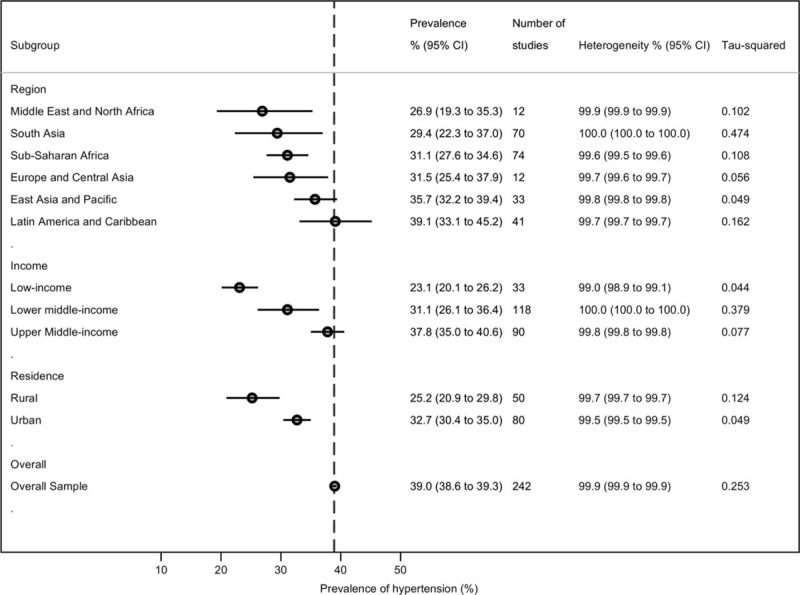
Country-specific hypertension prevalence.

The prevalence estimates of hypertension across regions, country income groups, and settings are shown in Figure 3. Subgroup analysis by region showed the highest prevalence estimate of hypertension in the Latin America and Caribbean region (39.1% [95% CI 33.1–45.2]) (eFigure 2, http://links.lww.com/MD/A543)^29,35,46–73^ whereas the Middle East and North Africa region had the lowest prevalence (26.9% [95% CI 19.3–35.3]) (eFigure 3, http://links.lww.com/MD/A543).^74–81^

The East Asia and Pacific region (35.7% [95% CI 32.2–39.4]) (eFigure 4, http://links.lww.com/MD/A543)^19–37^ and Europe and Central Asian region (32.0% [95% CI 28.0–37.0]) were second and third respectively to the Americas (eFigure 4, http://links.lww.com/MD/A543 and eFigure 5, http://links.lww.com/MD/A543). Prevalence estimates for the Sub-Saharan Africa^38–45^ and South Asia^29,35,82–108^ regions are shown respectively in eFigure 6, http://links.lww.com/MD/A543 and eFigure 7, http://links.lww.com/MD/A543.

### Pooled Prevalence by Country Income Groups

Upper middle income countries had a higher prevalence of hypertension (37.8% [95% CI 35.0–40.6]), compared with lower middle income (31.1% [95% CI 26.1–36.4]) and low-income countries (23.1% [95% CI 20.1–26.2]) (eFigures 8–10, http://links.lww.com/MD/A543). Hypertension prevalence was also higher among populations in urban settings (32.7% [95% CI 30.4–35.0]), compared with populations in rural settings (25.2% [95% CI 20.9–29.8]) (eFigures 11 and 12, http://links.lww.com/MD/A543).^29–31,40,42,76,78,80,99,111,113,121,128,131,135,136,140,146^

### Pooled Prevalence by Participants’ Socio-Demographic Characteristics

We summarized the patterns of hypertension across different socio-demographic characteristics for each region in Table 3. In all regions except the Middle East and North Africa region, where no data on hypertension prevalence was reported for the elderly (≥ 65 years), the proportion of hypertension was substantially higher among adults ≥ 65 years, compared to adults < 65 years (mean prevalence 65.6% [95% CI 53.6–75.0] vs 28.7% [95% CI 21.8–37.6], *P* < 0.00001).^22,23,30,31,40,42,46,55,56,59,61,62,65,66,69,70,84,90,91,95,97,99,101,113,117,121,122,128,131,134–136,146,147^ Nonetheless, hypertension prevalence in the Middle East and North Africa region was reportedly high among adults < 65 years (32.4% [95% CI 18.7–47.9]).^78,79^

**TABLE 3:**
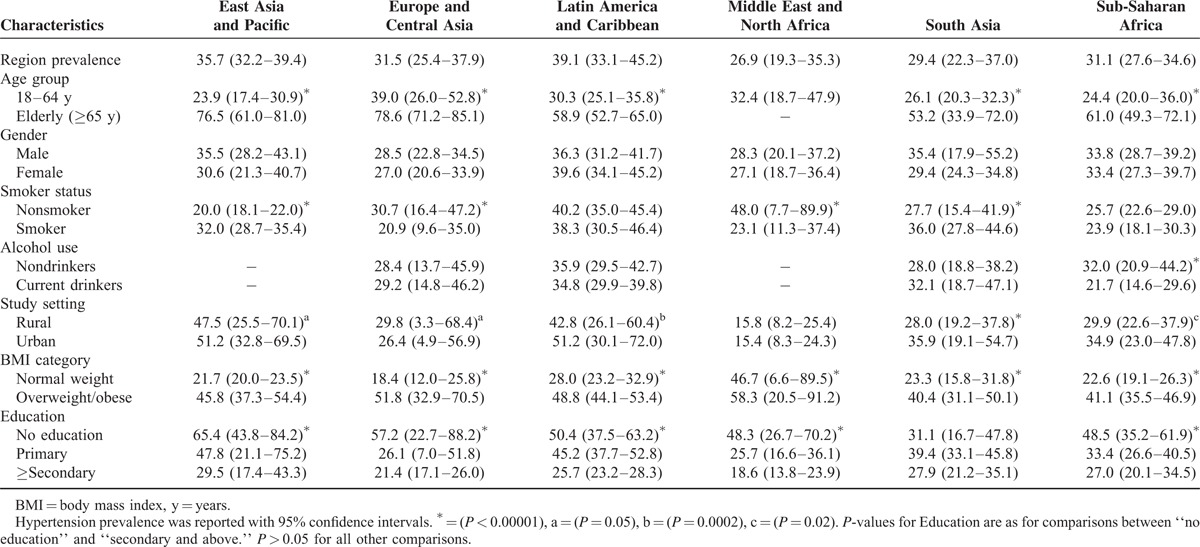
Prevalence Estimates of Hypertension by Region Across Socio-demographic Characteristics also Showing Risk of Bias in Included Studies

Although prevalence of hypertension in men overall (33%) was slightly higher compared with women (31.2%), this difference was not statistically significant (*P* = 0.76). Additionally, no significant sex-deference in hypertension prevalence was observed by region.

We found higher proportions of hypertension among nonsmokers compared to smokers in Europe and Central Asia, Latin America and Caribbean, and Middle East and North Africa regions. Whereas the proportions of hypertension was higher amongst smokers compared to nonsmokers in East Asia and Pacific, South Asia, and Sub-Saharan Africa regions. Alcohol use data was not reported in any study originating from countries in the Middle East and North Africa or the East Asia and Pacific regions. With the exception of the sub-Saharan Africa region, hypertension rates were comparable between nondrinkers and current drinkers; nondrinkers had a substantially higher proportion of hypertension compared to current drinkers in sub-Saharan Africa (32.0% [95% CI 20.9–44.2] vs 21.7% [95% CI 14.6–29.6], *P* < 0.00001).^113,121,131,135,136,140^ Importantly, hypertension rates were consistently higher among overweight/obese participants, compared to normal weight persons across all regions (mean prevalence 46.4% [95% CI 33.1–60.7] vs 26.3% [95% CI 15.8–37.8], *P* < 0.00001).^30,33,41,44,47,55,56,61,62,65,66,69,78,85,90,97,99,106,113,117,128,131,134–136,140^

With respect to study setting, prevalence estimates of hypertension were higher in urban communities, compared to participants in rural settings in the Latin America and Caribbean region (51.2% [95% CI 30.1–72.0] vs 42.8% [95% CI 26.1–60.4], *P* = 0.00017),^29^ East Asia and Pacific (51.2% [32.8–69.5] vs 47.5% [25.5–70.1], *P* < 0.00001), South Asia (35.9% [95% CI 19.1–54.7] vs 28.0% [95% CI 18.8–38.2], *P* < 0.00001),^29,99^ and Sub-Saharan Africa (34.9% [95% CI 23.0–47.8] vs 29.9% [95% CI 22.6–37.9], *P* = 0.017).^111,113,121,128,131,135,136,140,146^

Hypertension rates were also generally higher among the noneducated, compared to participants with a primary education (mean prevalence 50.2% [95% CI 30.4 – 69.3] vs 36.3% [95% CI 23.7–50.4], *P* < 0.00001) and participants with a secondary or tertiary education (mean prevalence 25.0% [95% CI 18.8 – 31.9], *P* < 0.00001).^22,30,31,33,40,41,44,47,55,56,61,65,66,69,70,76,79,81,97,99,106,113,117,121,128,131,134,136,140,146,147^

### Factors Associated With the Overall Hypertension Prevalence

**FIGURE 4:**
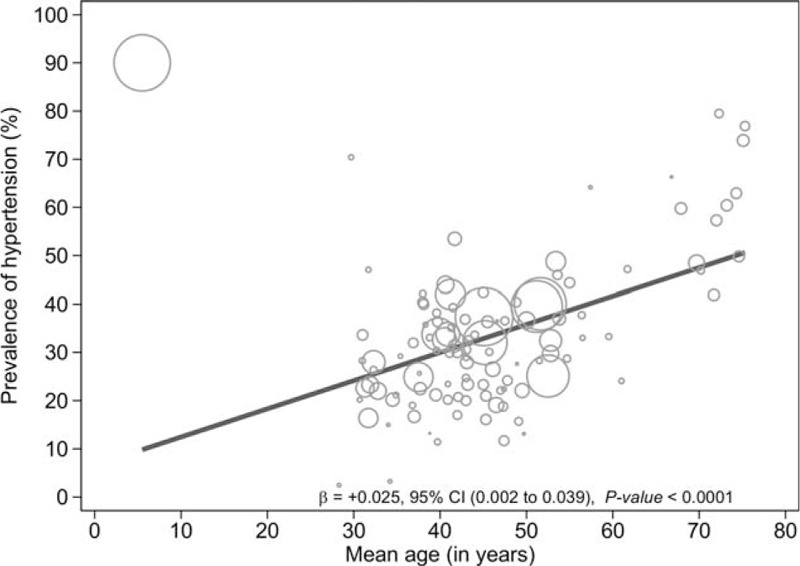
Meta-regression of hypertension against age.

**FIGURE 5:**
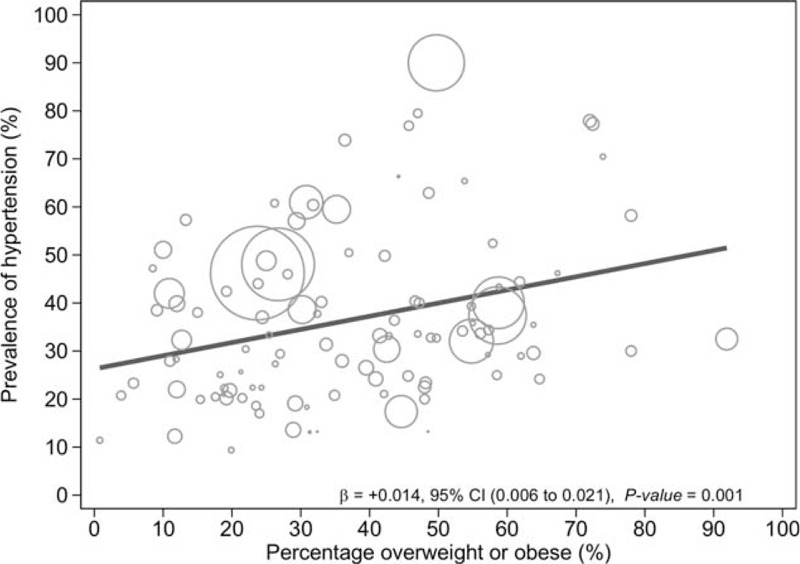
Meta-regression of hypertension prevalence against overweight/obesity.

**FIGURE 6:**
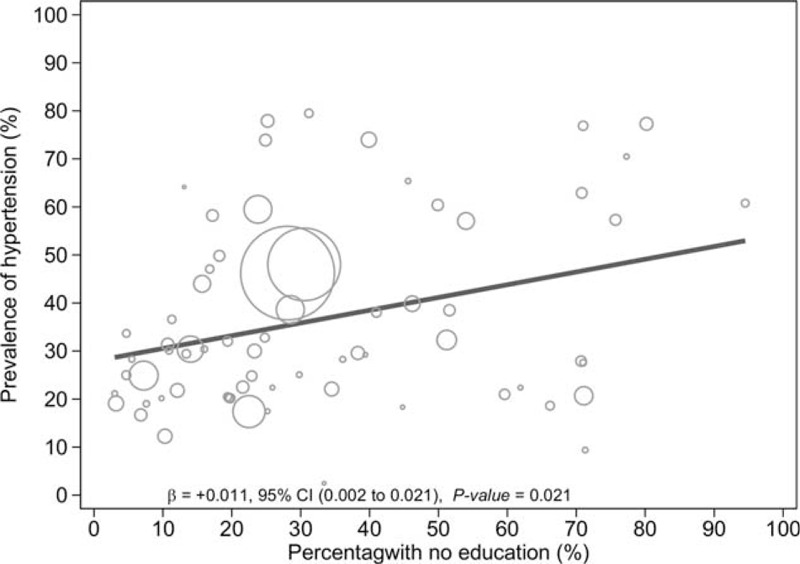
Meta-regression of hypertension against education.

The results of the meta-regression analyses showed that age (coefficient + 0.04 [95% CI 0.03–0.05], *P* < 0.0001) (Fig. 4), overweight/obesity (coefficient + 0.02 [95% CI 0.01–0.03] *P* = 0.001) (Fig. 5), and educational status (coefficient + 0.01 [95% CI 0.001–0.02] P = 0.049) (Fig. 6) accounted for significant heterogeneity in hypertension prevalence between studies. Given that <50% of the included studies had a low risk of sampling bias, we performed meta-regression analysis to determine if the observed heterogeneity in hypertension prevalence was partly influenced by variations in population sampling between studies; our analysis showed no statistically significant effect (coefficient + 0.11 [95% CI −0.04–0.26], *P* = 0.14).

## DISCUSSION

### Main Findings

To our knowledge, this is the first systematic review on hypertension prevalence and its socio-demographic patterning in the developing world. Our review suggests that hypertension remains an important public health problem in LMICs, with 1 in 3 persons affected by the disease. Our findings also suggest that older age and increased body weight may be consistent predictors of hypertension across LMICs, given that prevalence estimates of hypertension were substantially higher in the elderly, compared with younger adults, and in overweight/obese persons, compared with normal weight persons. Lower educational status was also found to be associated with a high prevalence of hypertension; however, this association was not consistent across counties and geographical regions.

Our results correspond to Kearney and colleagues^4^ who found hypertension prevalence to be highest among Latin America and Caribbean populations, compared to other regions, based on data collected prior to 2001. This broadly suggests that hypertension prevalence estimates in the Latin America and Caribbean region may have driven the burden of the disease in the developing world for more than a decade. Hypertension rates were more likely to be higher in upper middle income countries than in lower middle income countries, and the latter more likely to be higher compared to low-income countries. We hypothesize that a temporal relationship exists between increasing levels of affluence and urbanization and raised blood pressure. This plausibly accounts for the graded rise in hypertension rates, which is characteristic of the epidemiological transition within urban societies in resource-limited settings.^241,242^ The higher prevalence of hypertension in urban settings compared to rural settings, as shown in our study and in previous reviews,^6,243^ are also in accordance with this hypothesis.

Our findings also suggest that hypertension may be associated with socio-economic inequalities in LMICs: prevalence estimates for hypertension were inversely proportional to educational attainment, resulting in a downward socioeconomic gradient for hypertension. Evidence of health inequalities associated with hypertension in LMICs are not uncommon.^244,245^ Although men and women hypertension prevalence estimates were comparable overall, the slightly higher prevalence in men than women observed in our results are consistent with previous systematic reviews.^6,243^ This slight difference may be explained by evidence confirming male predilection for cardiovascular problems in the middle-age group, which attenuates in older adults as exemplified by an increased risk of cardiovascular diseases among post-menopausal women.^244,245^ Nonetheless, we observed a significant direct association between mean age and hypertension prevalence, which may be attributed to age-related structural changes in blood vessels which potentially cause narrowing of the vascular lumen, and consequently increasing blood pressure, as have been reported in previous studies.^246–248^ This hypothesis may also explain the unusually high prevalence estimates of hypertension (>70%) observed in a few of the included studies,^30,32,232^ given that the sampled population in these studies comprised mostly of the elderly.

Our review confirms that smoking is an independent risk factor for hypertension in Asian communities,^249,250^ given that smokers were more likely than nonsmokers to be hypertensive in the Asia regions alone.

Nevertheless, the higher proportions of hypertension among nonsmokers compared to smokers in the non-Asia regions may be attributed to smokers erroneously self-reporting their smoker status as nonsmokers in some of the included studies. Whereas this argument may account for the higher prevalence of hypertension among nondrinkers, compared to drinkers, in the sub-Saharan Africa region, it is also important to emphasize that nonsmokers and nondrinkers alike may engage in other harmful behaviors known to increase the risk of hypertension. Obviously, we cannot rule out the potential of reverse causation in cross-sectional studies, as it is possible that both nonsmokers and nondrinkers may have quit these harmful behaviors due to underlying medical conditions.^251^ However, it is important to state that we were not able to separate never smokers and never drinkers from exsmokers and exdrinkers in most of the included studies.

The association between combined overweight/obesity and hypertension shown in our results exemplify the role of excess body weight in hypertension prevalence, which has been long recognized and consistent across numerous observational and trial data.^252–256^

### Study Limitations and Strengths

We acknowledge that the overall quality of the included studies was moderate at best, especially given that more than a third of the studies (39%) were assessed as having high risk of sampling bias. However, as shown using meta-regression analysis, we emphasize that the high rates of sampling bias had no undue impact on the overall hypertension prevalence.

High amounts of heterogeneity across the included studies were another limitation of our study. Prevalence estimates from different regions were pooled in this meta-analysis, and as expected, high heterogeneity between studies was found in the meta-analyses. A substantial amount of the heterogeneity across studies was explained by such factors as differences in population characteristics and study methodologies. Nonetheless, as affirmed by previous evidence, meta-analyses are the preferred options to narrative syntheses for interpreting the results in a review, even in spite of the presence of a considerable amount of heterogeneity.^257^ Heterogeneity appeared to be the norm rather than exception in published meta-analyses of observational studies, in which case, it should be expected and quantified appropriately.^258^ Although we found some evidence of publication bias, it has been documented that tests of publication bias may lead to false-positive results in the presence of significant heterogeneity.^257,259^ Nonetheless, the “trim and fill” analysis revealed that the presence of publication bias had no significant impact on the overall prevalence of hypertension. Although we might have missed some potentially relevant studies; however, this systematic review arguably constitutes the largest study on hypertension prevalence in the resource-limited settings, comprising >1 million participants. In addition, we speculate that nonsmokers and nondrinkers may potentially be at risk of hypertension due to other health-damaging behaviors; our study therefore highlights the importance of expatiating on other lifestyle variables as potential correlates of hypertension and other cardiovascular conditions. For example, we were not able to examine the potential impact of differential dietary patterns and dietary salt intake on the observed variations in hypertension prevalence across countries. It is likely that differences in average dietary salt intake at the population level may contribute to some of the observed variations in hypertension prevalence across countries and world regions.^259–261^

In spite of the aforementioned limitations, the review's strengths are important. We conducted comprehensive searches of databases to ensure that all relevant publications were identified. We also reduced potential bias in the conduct of this review by having the authors independently scan through the search output and extract the data. In addition, there was reasonable coverage of evidence for most geographic regions, such as South Asia and sub-Saharan Africa; these regions were well represented by a sufficient number of studies with large sample size, which allows for generalizability of the results across these geographic regions.

### Implications of the Results

The elderly, overweight/obese, noneducated and urban settlers present opportunities for targeted health promotion and preventive interventions in LMICs. Given the high burden of infectious diseases in these countries, it might be economically justified to implement intervention programs for hypertension in higher-risk populations alone. However, the occurrence of hypertension in the general population remains unacceptably high, which poses an ethical dilemma to relying on high-risk strategies only in these settings; countries in the Middle East and North Africa region may even not have sufficient evidence to implement public health interventions in certain high-risk populations such as the elderly.

Health inequalities associated with hypertension have been recognized as an important public health issue in low- and middle-income countries.^244,245,262^ Addressing the wider social determinants of the disease is therefore crucial to its control in these countries. Failure to address these issues portends additional threats to the sustainability of public health infrastructure, especially alongside the prevailing effects of infectious disease epidemics.

Population-wide strategies such as reduction in dietary salt intake from processed foods are warranted in these low-resource settings, because they have been proven to be cost-effective means to shift blood pressure distribution at the population level, thus reducing the burden of cardiovascular disease associated with the epidemic of hypertension.^263–268^ Specifically, population-wide salt reduction through legislation, voluntary agreements with food industries and mass media campaigns are evidence-based cost-effective strategies for reducing hypertension prevalence in low- and middle-income countries, potentially preventing millions of years lost to the disease as a result of ill-health, disability or premature death.^269^ The absence of studies targeting the elderly in the Middle East and North Africa region also emphasizes the need for further research into resident high-risk subgroups. Hypertension among the elderly is likely to be a significant public health problem in the region considering that prevalence estimates in the young and middle-age are also high.

In conclusion, this study provides contemporary and up-to-date estimates that reflect the significant burden of hypertension and evidence that hypertension remains a major public health issue in LMICs. On average, about one-third of the adult population in these countries are hypertensive. However, this evidence originates from studies limited by high risk of selection bias and substantial between-study variations in the results. Nonetheless, we provide the most comprehensive evidence and first pooled analyses on hypertension prevalence in the developing world. There is a need for studies to accurately predict future trends of hypertension prevalence estimates in low- and middle-income countries. Additionally, future studies should explore alternative techniques to address heterogeneity, such as disease mapping or hierarchical modeling. The findings of this study would be useful for the design of hypertension screening and treatment programs in LMICs.

## Supplementary Material

Supplemental Digital Content
