# Supplementary material for: Prevalence of Hypertension in Low- and Middle-Income Countries: A Systematic Review and Meta-Analysis
Source: Medicine (Baltimore). 2015 Dec 18;94(50):e1959. doi: 10.1097/MD.0000000000001959 (PMC5058882; doi:10.1097/MD.0000000000001959)

APPENDICIES

Appendix 1: Search strategy

#1	‘hypertension’ or ‘blood pressure’ or ‘hypertens*’
#2	‘population based’ or ‘aetiology’ or etiology’ or ‘prevalence’ ‘epidemiolog*’or
#3	‘low-and middle-income countries’ or ‘developing countries’ or ‘Afghanistan ’or ‘Albania’ or ‘Algeria ’or ‘American Samoa’ or ‘Angola’ or ‘Antigua and Barbuda’ or ‘Argentina’ or ‘Armenia or ‘Azerbaijan’ or ‘Bangladesh’ or ‘Belarus’ or ‘Belize’ or ‘Benin’ or ‘Bhutan’ or ‘Bolivia’ or ‘Bosnia and Herzegovina’ or ‘Botswana’ or ‘Brazil’ or ‘Bulgaria’ or ‘Burkina Faso’ or ‘Burundi’ or ‘Cambodia’ or ‘Cameroon’ or ‘Cape Verde ’or ‘Central African Republic’ or ‘Chad’ or ‘China’ or ‘Colombia’ or ‘Comoros’ or ‘Congo Democratic Republic’ or ’Congo’ or ‘Costa Rica’ or ‘Cote d'Ivoire’ (Ivory Coast) or ‘Cuba’ or ‘Djibouti’ or ‘Dominica’ or ‘Dominican Republic’ or ‘ Egypt’ or ‘El Salvador’ or ‘Eritrea’ or ‘Ethiopia’ or ‘Fiji’ or ‘Gabon’ or ‘Gambia’ or ‘Georgia’ or ‘Ghana’ or ‘Grenada’ or ‘Guatemala’ or ‘Guinea’ or ‘Guinea-Bissau’ or ‘Guyana’ or ‘Haiti’ or ‘Honduras’ or ‘India’ or ‘Indonesia’ or ‘Iran Islamic Republic’ or ‘Iraq’ or ‘Jamaica’ or ‘Jordan’ or ‘Kazakhstan’ or ‘Kenya’ or ‘Kiribati’ or ‘Korea Democratic Republic’ or ‘Kosovo’ or ‘Kyrgyz Republic’ or ‘Lao Peoples Democratic Republic’ or ‘Lebanon’ or ‘Lesotho’ or ‘Liberia’ or ‘Libya’ or ‘Macedonia’ or ‘Madagascar’ or ‘Malawi’ or ‘Malaysia’ or ‘Maldives’ or ‘Mali’ or ‘Marshall Islands’ or ‘Mauritania’ or ‘Mauritius’ or ‘Mayotte’ or ‘Mexico’ or ‘Federated States of Micronesia’ or ‘Moldova’ or ‘Mongolia’ or ‘Montenegro’ or ‘Morocco’ or ‘Mozambique’ or ‘Myanmar’ or ‘Namibia’ or ‘Nepal’ or ‘Nicaragua’ or ‘Niger’ or ‘Nigeria’ or ‘Pakistan’ or ‘Palau’ or ‘Panama’ or ‘Papua New Guinea’ or ‘Paraguay’ or ‘Peru’ or ‘Philippines’ or ‘Romania’ or ‘Rwanda’ or ‘Samoa’ or ‘Sao Tome and Principe’ or ‘Senegal’ or ‘Serbia’ or ‘Seychelles’ or ‘Sierra Leone’ or ‘Solomon Islands’ or ‘Somalia’ or ‘South Africa’ or ‘Sri Lanka’ or ‘Saint Lucia’ or ‘Saint Vincent and the Grenadines’ or ‘Sudan’ or ‘Suriname’ or ‘Swaziland’ or ‘Syrian Arab Republic’ or ‘Tajikistan’ or ‘Tanzania’ or ‘Thailand’ or ‘Timor-Leste’ or ‘Togo’ or ‘Tonga’ or ‘Tunisia’ or ‘Turkey’ or ‘Turkmenistan’ or ‘Tuvalu’ or ‘Uganda’ or ‘Ukraine’ or ‘Uzbekistan’ or ‘Vanuatu’ or ‘Venezuela’ or ‘Vietnam’ or ‘West Bank and Gaza’ or ‘Yemen Republic’ or ‘Zambia’ or ‘Zimbabwe’.
#4	#1 and #2 and #3

Appendix 2: Risk of bias assessment tool (Newcastle-Ottawa scale)

**Domain (source of bias)**	**Assessment**	**Risk of bias**
Selection (representativeness of the sample)	All subjects or random sampling (A)	Low
	Non-random sampling (B)	Moderate
	Selected group of users (C)	High
	No description of sampling strategy (D)	Unclear/High
Selection (sample size)	Justified and satisfactory (A)	Low
	Not justified (B)	High
Detection (outcome measurement)	Validated measurement tool (A)	Low
	Tool described but non-validated (B)	High
	Tool not described (C)	Unclear/High
Confounding	Adjusted for confounders (A)	Low
	No adjustment for confounders (B)	High
(Detection) Outcome assessment	Independent blind assessment (A)	-
	Record linkage (B)	-
	Self-report (C)	-
	No description (D)	-

We first transformed the proportion of participants with hypertension in each study via the Freeman-Tukey double arcsine method[1] then performs an inverse-variance weighted random effects meta-analysis by conventional methods[2].

The pooled proportion can be calculated as the back-transform of the weighted mean of the transformed proportions:

$$\hat{p}=\frac{1}{2}\left[1-sign\left(\cos(\hat{x})\right)\sqrt{1-\left(\sin(\hat{x})+\frac{\sin(\hat{x})-\frac{1}{\sin(\hat{x})}}{n}\right)^{2}}\right]$$

$$\hat{x}=\frac{\sum xw}{\sum w}$$

$$x=\sin^{-1}\left(\sqrt{\frac{r}{n+1}}\right)+\sin^{-1}\left(\sqrt{\frac{r+1}{n+1}}\right)$$

$$w=n+0.5$$

$$q=\sum w\left(x-\hat{x}\right)^{2}$$

[1] Miller JJ. Inverse of the Freeman-Tukey Double Arcsine Transformation. The American Statistician 1978;32(4):138.
[2] DerSimonian R, Laird N. Meta-analysis in Clinical Trials. Controlled Clinical Trials 1986;7:177-188.

- where p hat is the fixed effects pooled proportion, x is the Freeman-Tukey transformed proportion, w is the inverse variance weight for the transformed proportion, q is the Cochran q statistic, tau squared is the moment-based estimate of the between-studies variance, w r is the DerSimonian-Laird weight, and p hat r is the random effects estimate of the pooled proportion.

Appendix 4: PRISMA checklist

Section/topic	#	Checklist item	Reported on page #
**TITLE**			
Title	1	Identify the report as a systematic review, meta-analysis, or both.	1
**ABSTRACT**			
Structured summary	2	Provide a structured summary including, as applicable: background; objectives; data sources; study eligibility criteria, participants, and interventions; study appraisal and synthesis methods; results; limitations; conclusions and implications of key findings; systematic review registration number.	2
**INTRODUCTION**			
Rationale	3	Describe the rationale for the review in the context of what is already known.	3
Objectives	4	Provide an explicit statement of questions being addressed with reference to participants, interventions, comparisons, outcomes, and study design (PICOS).	3
**METHODS**			
Protocol and registration	5	Indicate if a review protocol exists, if and where it can be accessed (e.g., Web address), and, if available, provide registration information including registration number.	4
Eligibility criteria	6	Specify study characteristics (e.g., PICOS, length of follow-up) and report characteristics (e.g., years considered, language, publication status) used as criteria for eligibility, giving rationale.	4
Information sources	7	Describe all information sources (e.g., databases with dates of coverage, contact with study authors to identify additional studies) in the search and date last searched.	5
Search	8	Present full electronic search strategy for at least one database, including any limits used, such that it could be repeated.	5
Study selection	9	State the process for selecting studies (i.e., screening, eligibility, included in systematic review, and, if applicable, included in the meta-analysis).	5
Data collection process	10	Describe method of data extraction from reports (e.g., piloted forms, independently, in duplicate) and any processes for obtaining and confirming data from investigators.	5-6
Data items	11	List and define all variables for which data were sought (e.g., PICOS, funding sources) and any assumptions and simplifications made.	5-6
Risk of bias in individual studies	12	Describe methods used for assessing risk of bias of individual studies (including specification of whether this was done at the study or outcome level), and how this information is to be used in any data synthesis.	6

Section/topic	#	Checklist item	Reported on page #
Summary measures	13	State the principal summary measures (e.g., risk ratio, difference in means).	6-7
Synthesis of results	14	Describe the methods of handling data and combining results of studies, if done, including measures of consistency (e.g., I^2) for each meta-analysis.	6-7
Risk of bias across studies	15	Specify any assessment of risk of bias that may affect the cumulative evidence (e.g., publication bias, selective reporting within studies).	6
Additional analyses	16	Describe methods of additional analyses (e.g., sensitivity or subgroup analyses, meta-regression), if done, indicating which were pre-specified.	6-7
**RESULTS**			
Study selection	17	Give numbers of studies screened, assessed for eligibility, and included in the review, with reasons for exclusions at each stage, ideally with a flow diagram.	6-7
Study characteristics	18	For each study, present characteristics for which data were extracted (e.g., study size, PICOS, follow-up period) and provide the citations.	6-8
Risk of bias within studies	19	Present data on risk of bias of each study and, if available, any outcome level assessment (see item 12).	8-9
Results of individual studies	20	For all outcomes considered (benefits or harms), present, for each study: (a) simple summary data for each intervention group (b) effect estimates and confidence intervals, ideally with a forest plot.	9-11
Synthesis of results	21	Present results of each meta-analysis done, including confidence intervals and measures of consistency.	9-11
Risk of bias across studies	22	Present results of any assessment of risk of bias across studies (see Item 15).	9
Additional analysis	23	Give results of additional analyses, if done (e.g., sensitivity or subgroup analyses, meta-regression [see Item 16]).	11-12
**DISCUSSION**			
Summary of evidence	24	Summarize the main findings including the strength of evidence for each main outcome; consider their relevance to key groups (e.g., healthcare providers, users, and policy makers).	12-14
Limitations	25	Discuss limitations at study and outcome level (e.g., risk of bias), and at review-level (e.g., incomplete retrieval of identified research, reporting bias).	15-16
Conclusions	26	Provide a general interpretation of the results in the context of other evidence, and implications for future research.	17-18

Section/topic	#	Checklist item	Reported on page #
**FUNDING**			
Funding	27	Describe sources of funding for the systematic review and other support (e.g., supply of data); role of funders for the systematic review.	18

Appendix 5: Risk of bias assessment for each study

Publication year	First Author	Country	Selection (sampling)	Selection (sample size)	Detection (outcome exposure)	Control confounders	Detection (outcome assessment)
1994	Gupta	India	D	A	C	A	D
1995	Gupta	India	A	A	A	A	D
1995	Ibrahim	Egypt	A	B	A	A	D
1996	Kaufman	Nigeria	A	A	A	A	D
1996	Goel	India	B	B	A	A	D
1998	Singh	India	A	B	A	A	D
1998	Ordunez-Garcia	Cuba	D	B	A	A	C
1999	Malhotra	India	A	A	A	A	D
2000	Edwards	Tanzania	A	B	C	A	D
2001	Barreto	Brazil	B	A	A	A	D
2001	Gurav	India	A	A	A	A	D
2001	Shmulewitz	Federated States of Micronesia	A	A	A	A	D
2001	Freitas	Brazil	A	B	A	A	D
2001	Wu	China	A	A	B	A	C
2002	Swami	India	A	B	A	A	D
2002	Hazarika	India	D	B	A	A	D
2002	Reddy	India	B	B	B	A	C
2002	Lorenzo	Mexico	B	A	D	B	D
2002	Gupta	India	B	A	A	A	D
2002	Gu	China	B	B	A	A	D
2003	Shapo	Albania	B	A	A	A	D
2003	Amoah	Ghana	C	B	A	A	D
2003	Matos	Brazil	A	A	C	A	D
2003	Bharucha	India	A	A	A	A	D
2003	Shanthirani	India	A	A	A	A	D
2003	Deepa	India	B	A	A	A	D
2003	Hazarika	India	D	B	A	A	D
2004	Onal	Turkey	A	B	A	A	D
2004	Cappuccio	Ghana	A	B	A	A	D
2004	Gus	Brazil	B	B	A	A	D
2004	Lim	Malaysia	A	A	A	A	D
2004	Gupta	India	C	B	A	A	D
2004	Hazarika	India	D	B	A	A	D
2005	Erhun	Nigeria	B	B	A	A	D
2005	Ordunez	Cuba	B	B	A	A	D
2005	Ahmad	Pakistan	B	A	C	A	C
2005	Siddiqui	Pakistan	A	A	A	A	D

Publication year	First Author	Country	Selection (sampling)	Selection (sample size)	Detection (outcome exposure)	Control confounders	Detection (outcome assessment)
2005	Das	India	A	B	A	A	D
2005	Prabhakaran	India	B	B	A	A	D
2006	Bahrami	Iran	A	A	A	A	D
2006	Agyemang	Ghana	B	B	A	A	D
2006	Lessa	Brazil	A	A	A	A	D
2006	Jean-Baptiste	Haiti	D	B	A	A	D
2006	Kamadjeu	Cameroon	A	A	A	A	D
2006	Minh	Vietnam	A	A	A	A	D
2006	Almeida-Pititto	Brazil	A	A	A	A	D
2006	Mishra	Uzbekistan	A	A	A	A	D
2006	Thankappan	India	A	A	A	A	D
2007	Jardim	Brazil	B	B	C	A	C
2007	Niakara	Burkina Faso	B	B	A	A	D
2007	Medina-Lezama	Peru	C	B	A	A	D
2007	Duda	Ghana	A	A	A	A	D
2007	Kengme	Cameroon	B	B	A	A	D
2007	Thorogood	South Africa	D	A	A	A	D
2007	Omuemu	Nigeria	A	B	A	A	D
2007	Vaidya	Nepal	B	A	A	A	D
2007	Mohan	India	A	A	A	A	D
2007	Wijewardene	SriLanka	B	A	A	A	D
2007	Reddyet	India	B	A	A	A	D
2007	Gupta	India	D	B	A	A	D
2007	Chaturvedi	India	A	B	B	A	D
2008	Rampal	Malaysia	D	B	A	A	D
2008	Ordunez	Cuba	C	B	B	A	D
2008	Capilheira	Brazil	A	B	A	A	D
2008	Sun	China	A	A	A	A	D
2008	Sparrenberger	Brazil	A	A	A	A	D
2008	Erem	Turkey	A	A	A	A	D
2008	Agrawal	India	D	B	A	A	D
2008	Yadav	India	D	B	A	A	D
2009	Azimi-Nezhad	Iran	A	A	A	A	D
2009	Ramezani	Iran	D	B	C	A	D
2009	Longo	Brazil	D	B	A	A	D
2009	Rosario	Brazil	B	A	A	A	D
2009	Zhang	China	A	B	B	A	D
2009	Rodrigues	Brazil	B	B	B	A	C
2009	Reichert	Brazil	B	B	B	A	C
2009	Diaz	Cuba	B	B	B	A	C
2009	Grimsrud	South Africa	B	B	B	A	C
2009	Wamala	Uganda	B	B	B	A	C

Publication year	First Author	Country	Selection (sampling)	Selection (sample size)	Detection (outcome exposure)	Control confounders	Detection (outcome assessment)
2009	Tesfaye	Ethiopia	B	B	B	A	C
2009	Damasceno	Mozambique	B	B	B	A	C
2009	Kusuma	India	B	B	B	A	C
2009	Pednekar	India	A	B	A	A	D
2009	Pednekar	India	B	A	A	A	D
2009	Midha	India	A	B	A	A	D
2010	Ebrahimi	Iran	B	B	A	A	D
2010	Cipullo	Brazil	A	B	C	A	D
2010	Nascente	Brazil	B	B	A	A	D
2010	Lee	China	B	B	A	A	D
2010	Swaddiwudhipong	Thailand	D	B	C	A	D
2010	Thuy	Vietnam	B	B	A	A	D
2010	Wu	China	D	B	A	A	D
2010	Dorobantu	Romania	D	B	A	A	D
2010	Ulasi	Nigeria	B	A	C	A	D
2010	Ekwunife	Nigeria	B	B	A	A	D
2010	Oladapo	Nigeria	B	B	A	A	D
2010	Sani	Nigeria	A	B	A	A	D
2010	Bhardwaj	India	D	B	A	A	D
2010	Jonas	India	A	A	C	A	D
2010	Kinra	India	A	B	A	A	D
2010	Kar	India	A	B	A	A	D
2011	Maher	Uganda	A	B	A	A	D
2011	Ulasi	Nigeria	A	B	A	A	D
2011	Wokoma	Nigeria	B	B	C	A	D
2011	Chataut	Nepal	D	B	A	A	D
2011	Norboo	India	D	B	C	A	D
2011	Manimunda	India	B	B	A	A	D
2011	Thrift	India	A	B	A	A	D
2012	Lyra	Brazil	A	B	C	A	D
2012	Berraho	Morocco	B	B	A	A	D
2012	Hofelmann	Brazil	A	B	A	A	D
2012	Kerkhoff	Brazil	A	A	A	A	D
2012	Altun	Turkey	A	A	A	A	D
2012	Prince	Cuba	D	B	A	A	D
2012	Dogan	Turkey	C	B	A	A	D
2012	Prince	Dominican Republic	A	A	C	A	D
2012	Prince	Peru	A	B	A	A	D
2012	Prince	Venezuela	A	B	A	A	D
2012	Prince	Mexico	A	B	A	A	D
2012	Prince	China	A	B	A	A	D
2012	Macia	Senegal	A	B	A	A	D

Publication year	First Author	Country	Selection (sampling)	Selection (sample size)	Detection (outcome exposure)	Control confounders	Detection (outcome assessment)
2012	Awoke	Ethiopia	B	A	A	A	D
2012	Mayega	Uganda	B	B	A	A	D
2012	Msyamboza	Malawi	A	B	C	A	D
2012	Hendriks	Nigeria	B	B	A	A	D
2012	Hendriks	Kenya	D	A	A	A	D
2012	Hendriks	Tanzania	D	B	A	A	D
2012	Hendriks	Namibia	D	B	A	A	D
2012	Dzudie	Cameroon	B	B	C	A	D
2012	Oladimeji	Nigeria	A	A	A	A	D
2012	Bharati	India	D	B	A	A	D
2012	Bansal	India	A	A	A	A	D
2012	Vaidya	Nepal	D	B	A	A	D
2012	Vaidya	Nepal	A	A	A	A	D
2012	Esam	India	D	B	A	A	D
2012	Dutta	India	D	A	A	B	D
2012	Prasad	India	D	B	A	A	D
2012	Prince	India	D	B	C	B	D
2012	Meshram	India	A	B	A	B	D
2012	Samuel	India	A	B	C	A	D
2012	Kaur	India	D	B	A	A	D
2012	Jeemon	India	A	B	A	A	D
2012	Gupta	India	D	A	A	A	D
2012	Gupta	India	A	A	A	B	D
2012	Chinnakali	India	B	B	A	B	D
2012	Kokiwar	India	A	B	A	A	D
2012	Borah	India	A	B	A	B	D
2012	Kaur	India	B	B	A	B	D
2013	Modesti	Yemen	A	B	A	A	D
2013	Harhay	Albania	B	B	A	A	D
2013	Harhay	Armenia	A	B	A	B	D
2013	Harhay	Azerbaijan	A	B	A	A	D
2013	Mendes	Brazil	B	B	A	A	D
2013	Selem	Brazil	A	A	C	A	D
2013	Ha	Vietnam	A	B	A	A	D
2013	Kiau	Malaysia	A	B	A	A	D
2013	Silva	Brazil	A	B	A	A	D
2013	Harhay	Ukraine	A	B	A	A	D
2013	Ogah	Nigeria	A	B	A	A	D
2013	Pessinaba	Senegal	B	A	A	A	D
2013	Peltzer	South Africa	B	B	A	A	D
2013	Kandala	South Africa	A	B	C	A	D
2013	Asekun-Olarinmoye	Nigeria	B	B	A	A	D

Publication year	First Author	Country	Selection (sampling)	Selection (sample size)	Detection (outcome exposure)	Control confounders	Detection (outcome assessment)
2013	Okpechi	Nigeria	D	A	A	A	D
2013	Veghari	Iran	D	B	A	A	D
2013	Ogunmola	Nigeria	D	B	A	A	D
2013	Ekanem	Nigeria	B	B	C	A	D
2013	Adebayo	Nigeria	A	A	A	A	D
2013	Peer	South Africa	D	B	A	A	D
2013	Musinguzi	Uganda	A	A	A	A	D
2013	Bhagyalaxmi	India	D	B	A	A	D
2013	Gupta	India	A	A	A	A	D
2013	Khan	Nepal	D	B	A	A	D
2014	Wang	China	D	A	A	B	D
2014	Feng	China	D	B	A	A	D
2014	Lloyd-Sherlock	China	D	B	C	B	D
2014	Fan	China	A	B	A	B	D
2014	Posso	Panama	A	B	C	A	D
2014	Awosan	Nigeria	D	B	A	A	D
2014	Lloyd-Sherlock	Ghana	A	B	A	A	D
2014	Lloyd-Sherlock	South Africa	D	A	A	A	D
2014	Helelo	Ethiopia	A	A	A	B	D
2014	Moges	Ethiopia	B	B	A	B	D
2014	Duboz	Senegal	A	B	A	A	D
2014	Doulougou	Burkina Faso	A	B	A	B	D
2014	Awuah	Ghana	B	B	A	B	D
2014	Doulougou	Burkina Faso	A	B	A	A	D
2014	Oluyombo	Nigeria	B	B	A	A	D
2014	Nguyen	Vietnam	A	B	A	B	D
2014	Lloyd-Sherlock	Mexico	A	B	A	A	D
2014	Amiri	Malaysia	B	B	A	A	D
2014	Zhao	China	A	A	C	A	D
2014	Adhikari	Nepal	A	B	A	A	D
2014	Lloyd-Sherlock	India	A	B	A	A	D
2014	Zaman	Bangladesh	A	B	A	A	D
2015	Abebe	Ethiopia	A	B	A	A	D
2015	Akpan	Nigeria	A	B	A	A	D
2015	Angaw	Ethiopia	B	A	A	A	D
2015	Bernabe Ortiz	Peru	B	B	A	A	D
2015	Bresan	Brazil	A	B	C	A	D
2015	De Souza	Brazil	B	B	A	A	D
2015	Anteneh	Ethiopia	D	A	A	A	D
2015	Asiki	Uganda	D	B	A	A	D
2015	Isara	Nigeria	D	B	A	A	D
2015	Muusinguzi	Uganda	B	B	C	A	D

Publication year	First Author	Country	Selection (sampling)	Selection (sample size)	Detection (outcome exposure)	Control confounders	Detection (outcome assessment)
2015	Khalifeh	Lebanon	A	A	A	A	D
2015	Matar	Lebanon	D	B	A	A	D
2015	Minicuci	Ghana	A	A	A	A	D
2015	Sepanlou	Iran	D	B	A	A	D
2015	Supiyev	Kazakhstan	A	A	A	A	D
2015	Oguoma	Nigeria	D	B	A	A	D
2015	Wandera	Uganda	D	A	A	B	D
2015	Hou	China	D	B	A	A	D
2015	Chen	China	D	B	C	B	D
2015	Lim	China	A	B	A	B	D
2015	Iazdanpanah	Iran	A	B	C	A	D
2015	Wang	China	D	B	A	A	D
2015	Sowemimo	Nigeria	A	B	A	A	D
2015	Guo	China	D	A	A	A	D
2015	Kingue	Cameroon	A	A	A	B	D
2015	Bushara	Sudan	B	B	A	B	D
2015	Seck	Senegal	A	B	A	A	D
2015	Ezeala-Adekaibe	Nigeria	A	B	A	B	D
2015	Li	China	B	B	A	B	D
2015	Unger	Brazil	A	B	A	A	D
2015	Ibekwe	Nigeria	B	B	A	A	D
2015	Ugwuja	Nigeria	A	B	A	A	D
2015	Botha	South Africa	A	A	A	A	D
2015	Vieira	Brazil	A	A	A	A	D
2015	Gu	China	A	A	A	A	D
2015	Almeida	Brazil	D	B	A	A	D
2015	Sander	Uganda	D	B	A	A	D
2015	Lu	China	A	A	A	A	D
2015	Ke	China	D	B	C	A	D
2015	Wei	China	D	B	A	A	D
2015	Do	Vietnam	B	A	A	A	D
2015	Gupta	India	A	B	B	A	D
2015	Menon	India	B	B	B	A	C
2015	Ranasighe	Sri Lanka	B	B	B	A	C
2015	Rahman	Bangladesh	B	B	B	A	C
2015	Bhansali	India	B	B	B	A	C

eTable 1: Characteristics of included studies for the East Asia and Pacific region

First author, year	Year of data collection	Country	Setting	Age (years)	Mean age ± SD (years)	Sample size	% Males	Hypertension prevalence (male/female)	BP cut-off (mmHg)	Country income group
Shmulewitz, 2001[19]	1994	FS Micronesia	rural	>20	42.0±14.0	2188	42.0	17.0	140/90	lower-middle
Wu, 2001[20]	2001	China	urban	≥60	67.9±5.8	2272	41.5	59.8	140/90	upper-middle
Gu, 2002[21]	2000-01	China	urban and rural	35-74	NR	13198	47.8	27.2(28.6/25.8)	140/90	upper-middle
Lim, 2004[22]	1996	Malaysia	urban and rural	≥30	NR	21391	47.0	33.0(31.9/33.9)	140/90	upper-middle
Minh, 2006[23]	2002	Vietnam	rural	25-64	NR	1996	50.1	14.1(18.1/10.1)	140/90	lower-middle
Rampal, 2008[24]	2004	Malaysia	urban and rural	≥15	NR	16440	42.4	27.8(29.6/26.0)	140/90	upper-middle
Sun, 2008[25]	2004-06	China	urban	≥35	51.2±11.8	45390	49.6	39.5(37.0/38.6)	140/90	upper-middle
Zhang, 2009[26]	2006	China	urban	≥60	69.7±6.7	4141	35.3	48.5(48.4/48.6)	140/90	upper-middle
Lee, 2010[27]	2002-06	China	urban	40-74	52.5±8.9	39252	100	25.1	140/90	upper-middle
Swaddiwudhipong, 2010[28]	2009	Thailand	rural	≥15	52.8±11.9	5273	44.9	29.8(29.8/29.7)	140/90	upper-middle
Thuy, 2010[29]	2005	Vietnam	urban	25-64	41.3±1.0	910	100	35.2	140/90	lower-middle
Wu, 2010[20]	2010	China	urban	≥60	71.7±6.6	2074	41	70.4	140/90	upper-middle
Prince, 2012[30]	2003-06	China	urban and rural	≥65	73.2±6.1	2157	43.7	60.4	140/90	upper-middle
Ha, 2013[31]	2011	Vietnam	urban and rural	≥25	45.0	2368	43.5	23.3(30.0/19.0)	140/90	lower-middle
Kiau, 2013[32]	2006	Malaysia	urban and rural	≥18	NR	4933	46.3	74.0(70.1/77.4)	140/90	upper-middle
Amiri, 2014[33]	2012	Malyasia	urban	≥18	41.5±14.9	1096	43.7	39.3	140/90	upper-middle
Fan, 2014[34]	2010	China	urban and rural	15-74	38.2±15.1	18772	40.4	24.9	140/90	upper-middle
Feng, 2014[35]	2011-12	China	urban and rural	≥45	NR	13707	46.7	38.6	140/90	upper-middle
Lloyd-Sherlock, 2014[36]	2007-10	China	urban and rural	≥50	NR	13348	49.8	59.5(58.8/60.1)	140/90	upper-middle
Nguyen, 2014[37]	2012	Vietnam	urban and rural	34-65	47.4±8	3779	43.3	12.3(28.3/14.6)	140/90	upper-middle
Wang, 2014[38]	2003-10	China	urban	≥18	45.2±13.9	37141	60.7	32.1(33.7/19.3)	140/90	upper-middle
Zhao, 2015[39]	2008-12	China	rural	18-60	53.4±10.3	6324	100	48.8	140/90	upper-middle
Hou, 2015[40]	2008-12	China	urban and rural	≥45	NR	3797	47.7	46.2	140/90	upper-middle
Chen, 2015[41]	2012	China	rural	≥50	NR	2208	43.1	38.5	140/90	upper-middle
Li, 2015[42]	2015	China	urban and rural	≥18	45.1±3.6	58985	43.2	37.4	140/90	upper-middle
Wang, 2015[43]	2009	China	urban and rural	18-87	41.2±15.5	15172	45.7	42	140/90	upper-middle

First author, year | Year of data collection | Country | Setting | Age (years) | Mean age ± SD (years) | Sample size | % Males | Hypertension prevalence (male/female) | BP cut-off (mmHg) | Country income group

Guo, 2015[44]	1991-2011	China	urban and rural	≥18	NR	24410	-	28.6	140/90	upper-middle
Li, 2015[45]	2015	China	urban	≥18	NR	2026	-	26.9	140/90	upper-middle
Gu, 2015[46]	2002	China	urban and rural	35-70	NR	7137	-	40.9	140/90	upper-middle
Lu, 2015[47]	2013	China	urban	≥18	45	4675	-	31.6	140/90	upper-middle
Ke, 2015[48]	2012	China	urban	18-93	NR	1410	45.2	34	140/90	upper-middle
Wei, 2015[49]	2015	China	urban	≥18	NR	3778	47.3	41	140/90	upper-middle
Do, 2015[50]	2005	Vietnam	urban and rural	25-64	NR	17199	-	20.7	140/90	lower-middle

BP=blood pressure, FS Micronesia=Federal States of Micronesia, SD=standard deviation, mmHg=millimetre mercury, NR=not reported in original paper.
Country income group is classified according to World Bank indicators

eFigure 1: Contour-enhanced funnel plot

eFigure 2: Prevalence estimate of hypertension in the Latin America and Caribbean region

eFigure 3: Prevalence estimate of hypertension in the Middle East and North Africa region

eFigure 4: Prevalence estimate of hypertension in the East Asia and Pacific region

eFigure 5: Prevalence estimate of hypertension in the Sub-Saharan Africa region

eFigure 6: Prevalence estimate of hypertension in the Europe and Central Asia region

eFigure 7: Prevalence estimate of hypertension in the South Asia region

eFigure 8: Prevalence estimate of hypertension in upper-middle income countries

eFigure 10: Prevalence estimate of hypertension in low-income countries

eFigure 11: Prevalence of hypertension in urban settings

eFigure 12: Prevalence of hypertension in rural settings

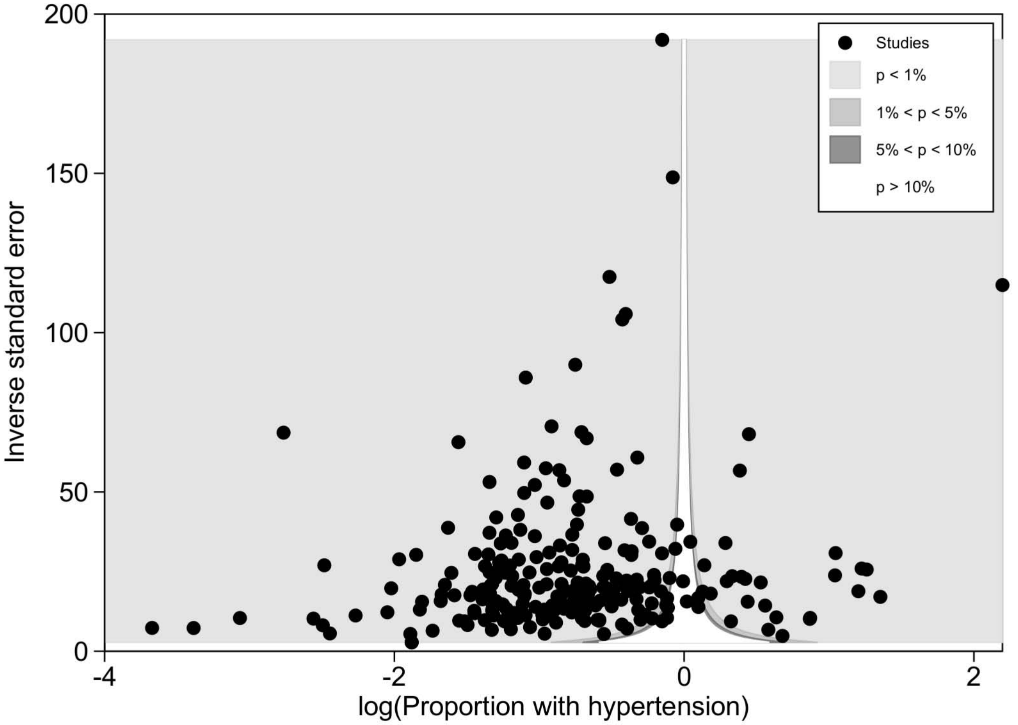

Studies
p < 1%
1% < p < 5%
5% < p < 10%
p > 10%
Inverse standard error
log(Proportion with hypertension)
200
150
100
50
0
-4
-2
0
2

Author (year)	Hypertension cases	Sample size	Prevalence of hypertension	95% CI
Ordunez−Garcia (1998)	719	1633	44.03	[41.60; 46.48]
Barreto (2001)	574	2314	24.81	[23.06; 26.62]
Freitas (2001)	217	688	31.54	[28.08; 35.16]
Lorenzo (2002)	429	2282	18.80	[17.22; 20.46]
Matos (2003)	46	126	36.51	[28.12; 45.55]
Gus (2004)	358	1063	33.68	[30.84; 36.61]
Ordunez (2005)	332	1667	19.92	[18.02; 21.92]
Almeida−Pititto (2006)	160	484	33.06	[28.88; 37.45]
Jean−Baptiste (2006)	524	1113	47.08	[44.11; 50.06]
Lessa (2006)	430	1439	29.88	[27.53; 32.32]
Jardim (2007)	633	1739	36.40	[34.13; 38.71]
Medina−Lezama (2007)	295	1878	15.71	[14.09; 17.43]
Capilheira (2008)	725	3100	23.39	[21.91; 24.92]
Ordunez (2008)	316	1475	21.42	[19.35; 23.61]
Sparrenberger (2008)	600	1484	40.43	[37.92; 42.98]
Diaz (2009)	11907	19519	61.00	[60.31; 61.69]
Longo (2009)	681	2022	33.68	[31.62; 35.79]
Reichert (2009)	583	1696	34.38	[32.11; 36.69]
Rodrigues (2009)	702	1655	42.42	[40.02; 44.84]
Rosario (2009)	302	1003	30.11	[27.28; 33.05]
Cipullo (2010)	762	1717	44.38	[42.01; 46.77]
Nascente (2010)	382	1168	32.71	[30.02; 35.48]
Hofelmann (2012)	688	1720	40.00	[37.67; 42.36]
Kerkhoff (2012)	635	1858	34.18	[32.02; 36.38]
Lyra (2012)	127	198	64.14	[57.04; 70.82]
Prince (2012)	2176	2944	73.91	[72.29; 75.49]
Prince (2012)	1538	2000	76.90	[74.99; 78.73]
Prince (2012)	961	1929	49.82	[47.56; 52.07]
Prince (2012)	1259	2001	62.92	[60.76; 65.04]
Prince (2012)	1422	1789	79.49	[77.54; 81.34]
Mendes (2013)	395	842	46.91	[43.50; 50.35]
Selem (2013)	232	535	43.36	[39.12; 47.68]
Silva (2013)	695	1720	40.41	[38.08; 42.77]
Lloyd−Sherlock (2014)	1328	2281	58.22	[56.16; 60.25]
Posso (2014)	1008	3406	29.59	[28.07; 31.16]
Almeida (2015)	750	1410	53.19	[50.55; 55.82]
Bernabe Ortiz (2015)	518	3218	16.10	[14.84; 17.41]
Bresan (2015)	164	355	46.20	[40.92; 51.54]
De Souza (2015)	432	1729	24.99	[22.96; 27.10]
Unger (2015)	1162	5649	20.57	[19.52; 21.65]
Vieira (2015)	111	491	22.61	[18.98; 26.57]
**Random effects model**		**87340**	**39.06**	**[33.12; 45.17]**

Heterogeneity: I−squared=99.7% [99.7%; 99.7%], tau−squared=0.162, p<0.0001

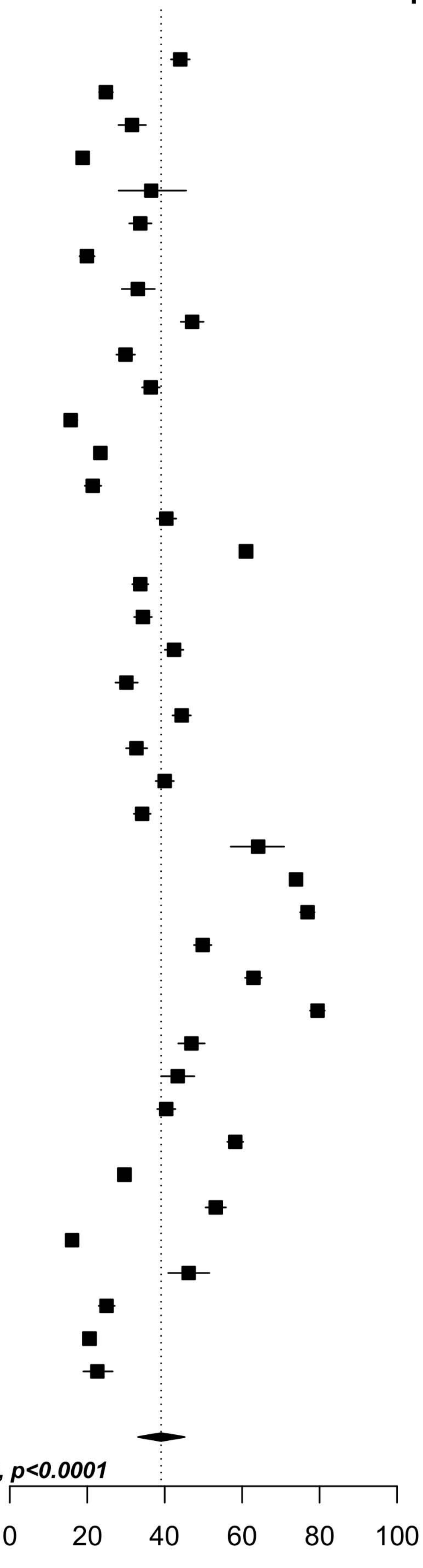

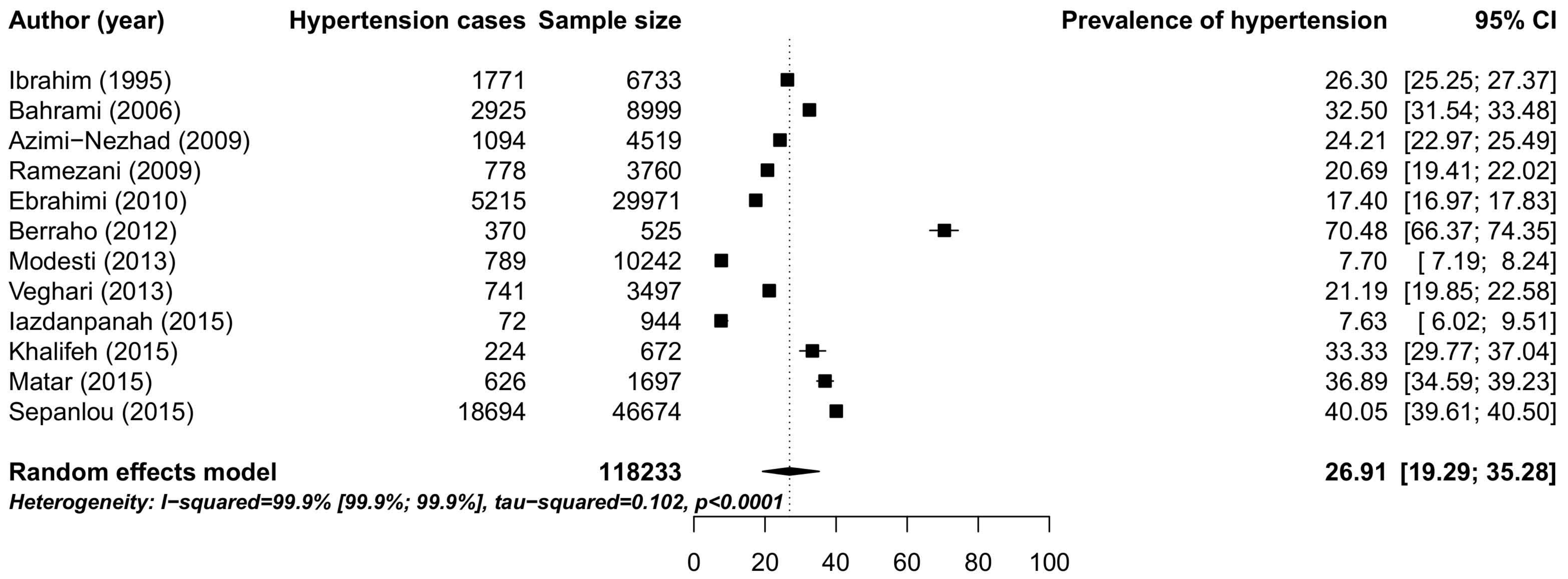
Author (year)
Hypertension cases
Sample size
Prevalence of hypertension
95% CI
Ibrahim (1995) 1771 6733 26.30 [25.25; 27.37]
Bahrami (2006) 2925 8999 32.50 [31.54; 33.48]
Azimi−Nezhad (2009) 1094 4519 24.21 [22.97; 25.49]
Ramezani (2009) 778 3760 20.69 [19.41; 22.02]
Ebrahimi (2010) 5215 29971 17.40 [16.97; 17.83]
Berraho (2012) 370 525 70.48 [66.37; 74.35]
Modesti (2013) 789 10242 7.70 [7.19; 8.24]
Veghari (2013) 741 3497 21.19 [19.85; 22.58]
Iazdanpanah (2015) 72 944 7.63 [6.02; 9.51]
Khalifeh (2015) 224 672 33.33 [29.77; 37.04]
Matar (2015) 626 1697 36.89 [34.59; 39.23]
Sepanlou (2015) 18694 46674 40.05 [39.61; 40.50]
Random effects model 118233 26.91 [19.29; 35.28]
Heterogeneity: I−squared=99.9% [99.9%; 99.9%], tau−squared=0.102, p<0.0001
0
20
40
60
80
100

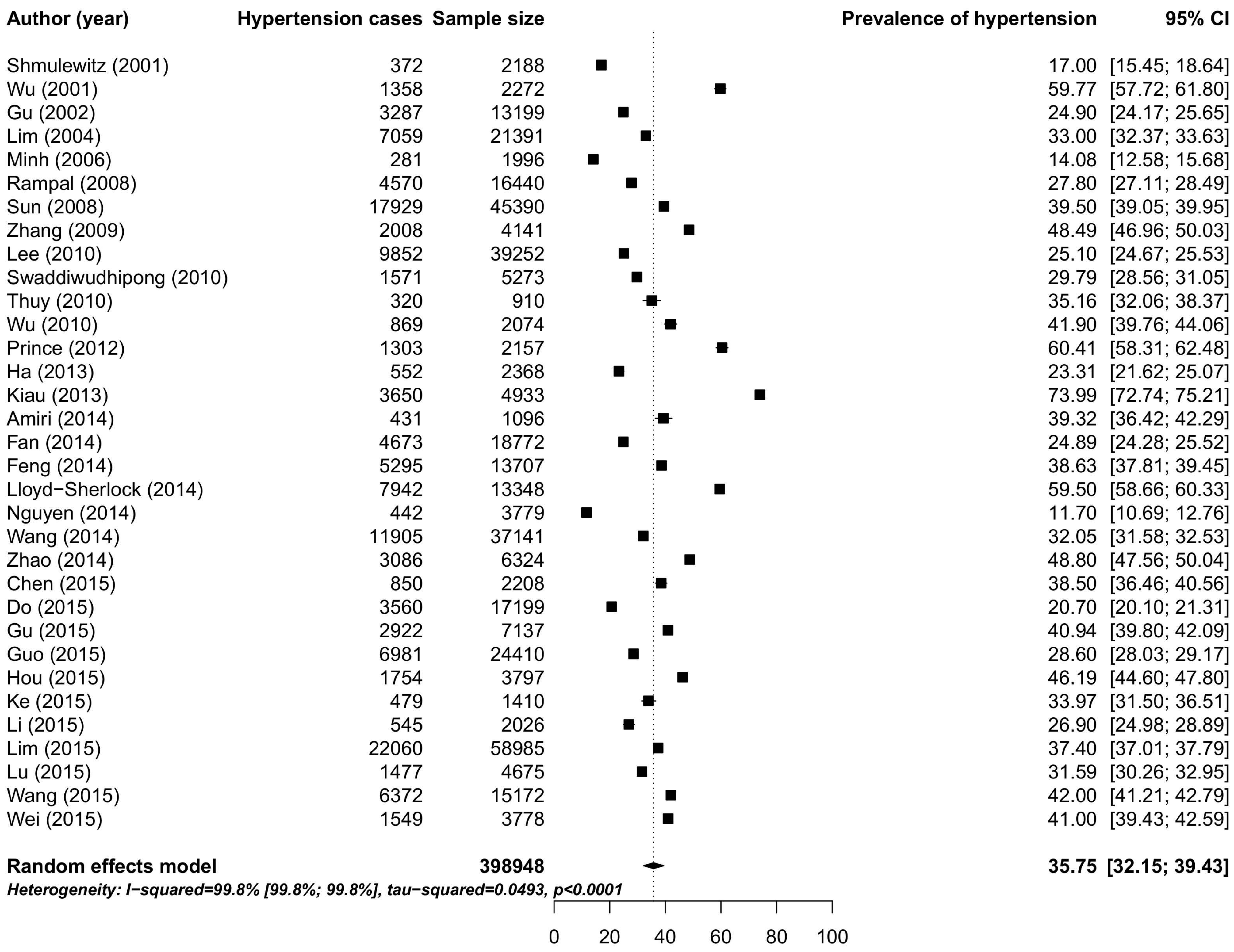
Author (year) Hypertension cases Sample size Prevalence of hypertension 95% CI
Shmulewitz (2001) 372 2188 17.00 [15.45; 18.64]
Wu (2001) 1358 2272 59.77 [57.72; 61.80]
Gu (2002) 3287 13199 24.90 [24.17; 25.65]
Lim (2004) 7059 21391 33.00 [32.37; 33.63]
Minh (2006) 281 1996 14.08 [12.58; 15.68]
Rampal (2008) 4570 16440 27.80 [27.11; 28.49]
Sun (2008) 17929 45390 39.50 [39.05; 39.95]
Zhang (2009) 2008 4141 48.49 [46.96; 50.03]
Lee (2010) 9852 39252 25.10 [24.67; 25.53]
Swaddiwudhipong (2010) 1571 5273 29.79 [28.56; 31.05]
Thuy (2010) 320 910 35.16 [32.06; 38.37]
Wu (2010) 869 2074 41.90 [39.76; 44.06]
Prince (2012) 1303 2157 60.41 [58.31; 62.48]
Ha (2013) 552 2368 23.31 [21.62; 25.07]
Kiau (2013) 3650 4933 73.99 [72.74; 75.21]
Amiri (2014) 431 1096 39.32 [36.42; 42.29]
Fan (2014) 4673 18772 24.89 [24.28; 25.52]
Feng (2014) 5295 13707 38.63 [37.81; 39.45]
Lloyd−Sherlock (2014) 7942 13348 59.50 [58.66; 60.33]
Nguyen (2014) 442 3779 11.70 [10.69; 12.76]
Wang (2014) 11905 37141 32.05 [31.58; 32.53]
Zhao (2014) 3086 6324 48.80 [47.56; 50.04]
Chen (2015) 850 2208 38.50 [36.46; 40.56]
Do (2015) 3560 17199 20.70 [20.10; 21.31]
Gu (2015) 2922 7137 40.94 [39.80; 42.09]
Guo (2015) 6981 24410 28.60 [28.03; 29.17]
Hou (2015) 1754 3797 46.19 [44.60; 47.80]
Ke (2015) 479 1410 33.97 [31.50; 36.51]
Li (2015) 545 2026 26.90 [24.98; 28.89]
Lim (2015) 22060 58985 37.40 [37.01; 37.79]
Lu (2015) 1477 4675 31.59 [30.26; 32.95]
Wang (2015) 6372 15172 42.00 [41.21; 42.79]
Wei (2015) 1549 3778 41.00 [39.43; 42.59]
Random effects model 398948 35.75 [32.15; 39.43]
Heterogeneity: I−squared=99.8% [99.8%; 99.8%], tau−squared=0.0493, p<0.0001
0 20 40 60 80 100

Author (year)	Hypertension cases	Sample size	Prevalence of hypertension	95% CI
Kaufman (1996)	144	598	24.08	[20.70; 27.71]
Edwards (2000)	520	1698	30.62	[28.44; 32.88]
Amoah (2003)	1339	4733	28.29	[27.01; 29.60]
Cappuccio (2004)	291	1013	28.73	[25.96; 31.62]
Erhun (2005)	210	1000	21.00	[18.51; 23.66]
Agyemang (2006)	421	1431	29.42	[27.07; 31.86]
Kamadjeu (2006)	2413	10011	24.10	[23.27; 24.95]
Duda (2007)	721	1321	54.58	[51.85; 57.29]
Kengme (2007)	532	2559	20.79	[19.23; 22.41]
Niakara (2007)	822	2044	40.22	[38.08; 42.38]
Omuemu (2007)	119	590	20.17	[17.00; 23.64]
Thorogood (2007)	171	402	42.54	[37.65; 47.53]
Damasceno (2009)	1100	3323	33.10	[31.50; 34.73]
Grimsrud (2009)	727	4351	16.71	[15.61; 17.85]
Tesfaye (2009)	1114	3713	30.00	[28.53; 31.51]
Wamala (2009)	256	842	30.40	[27.31; 33.63]
Ekwunife (2010)	160	756	21.16	[18.30; 24.25]
Oladapo (2010)	415	2000	20.75	[18.99; 22.59]
Sani (2010)	77	300	25.67	[20.82; 31.00]
Ulasi (2010)	478	1458	32.78	[30.38; 35.26]
Maher (2011)	1469	6678	22.00	[21.01; 23.01]
Ulasi (2011)	290	688	42.15	[38.43; 45.94]
Wokoma (2011)	42	152	27.63	[20.70; 35.46]
Awoke (2012)	192	679	28.28	[24.92; 31.83]
Dzudie (2012)	1007	2120	47.50	[45.36; 49.65]
Hendriks (2012)	555	1733	32.03	[29.83; 34.28]
Hendriks (2012)	426	2111	20.18	[18.49; 21.96]
Hendriks (2012)	562	2678	20.99	[19.46; 22.58]
Hendriks (2012)	199	1046	19.02	[16.69; 21.54]
Macia (2012)	327	500	65.40	[61.05; 69.57]
Mayega (2012)	339	1656	20.47	[18.55; 22.50]
Msyamboza (2012)	1237	3727	33.19	[31.68; 34.73]
Oladimeji (2012)	232	801	28.96	[25.84; 32.24]
Adebayo (2013)	264	1000	26.40	[23.69; 29.25]
Asekun−Olarinmoye (2013)	34	259	13.13	[9.27; 17.86]
Ekanem (2013)	208	442	47.06	[42.33; 51.83]
Kandala (2013)	4133	13596	30.40	[29.63; 31.18]
Musinguzi (2013)	995	4563	21.81	[20.62; 23.03]
Ogah (2013)	1566	2928	53.48	[51.66; 55.30]
Ogunmola (2013)	69	104	66.35	[56.42; 75.32]
Okpechi (2013)	936	2983	31.38	[29.71; 33.08]
Peer (2013)	460	1099	41.86	[38.92; 44.84]
Peltzer (2013)	2968	3840	77.29	[75.93; 78.61]
Pessinaba (2013)	655	1424	46.00	[43.38; 48.63]
Awosan (2014)	114	390	29.23	[24.76; 34.02]
Awuah (2014)	202	714	28.29	[25.01; 31.75]
Doulougou (2014)	139	1481	9.39	[7.95; 10.99]
Doulougou (2014)	380	2041	18.62	[16.95; 20.38]
Duboz (2014)	165	600	27.50	[23.96; 31.26]
Helelo (2014)	116	518	22.39	[18.87; 26.23]
Lloyd−Sherlock (2014)	2692	4716	57.08	[55.66; 58.50]
Lloyd−Sherlock (2014)	2976	3820	77.91	[76.56; 79.21]
Moges (2014)	9	68	13.24	[6.23; 23.64]
Mooes (2014)	9	68	13.24	[6.23; 23.64]
Oluyombo (2014)	354	750	47.20	[43.58; 50.84]
Abebe (2015)	614	2200	27.91	[26.04; 29.83]
Akpan (2015)	595	1565	38.02	[35.61; 40.48]
Angaw (2015)	179	655	27.33	[23.95; 30.91]
Anteneh (2015)	170	678	25.07	[21.85; 28.51]
Asilu (2015)	950	7741	12.27	[11.55; 13.02]
Botha (2015)	191	429	44.52	[39.75; 49.37]
Bushara (2015)	420	1099	38.22	[35.33; 41.16]
Ezeala−Adekaibe (2015)	406	774	52.45	[48.87; 56.02]
Ibekwe (2015)	57	272	20.96	[16.28; 26.28]
Isara (2015)	319	845	37.75	[34.47; 41.12]
Kingue (2015)	4595	15470	29.70	[28.98; 30.43]
Minicuci (2015)	2413	4724	51.08	[49.64; 52.51]
Muusinguzi (2015)	895	4432	20.19	[19.02; 21.41]
Oguoma (2015)	151	422	35.78	[31.20; 40.56]
Sander (2015)	34	426	7.98	[5.59; 10.97]
Seck (2015)	147	627	23.44	[20.18; 26.96]
Sowemimo (2015)	267	806	33.13	[29.88; 36.50]
Ugwuja (2015)	62	267	23.22	[18.29; 28.76]
Wandera (2015)	376	2382	15.79	[14.34; 17.31]
**Random effects model**		**161930**	**31.05**	**[27.61; 34.60]**

Heterogeneity: I−squared=99.6% [99.5%; 99.6%], tau−squared=0.108, p<0.0001

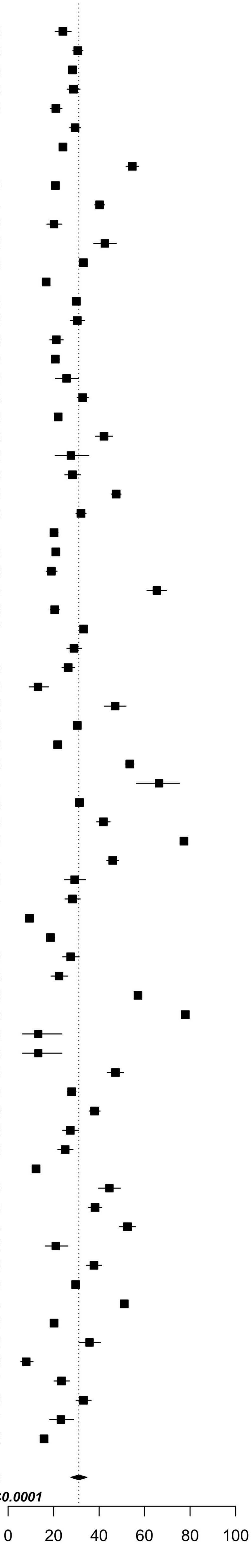

Author (year)	Hypertension cases	Sample size	Prevalence of hypertension	95% CI
Shapo (2003)	356	1120	31.79	[29.06; 34.60]
Onal (2004)	150	423	35.46	[30.90; 40.23]
Mishra (2006)	1060	7796	13.60	[12.84; 14.38]
Erem (2008)	2116	4809	44.00	[42.59; 45.42]
Dorobantu (2010)	906	2017	44.92	[42.73; 47.12]
Altun (2012)	3515	10748	32.70	[31.82; 33.60]
Dogan (2012)	492	2035	24.18	[22.33; 26.10]
Harhay (2013)	3025	10802	28.00	[27.16; 28.86]
Harhay (2013)	1707	7552	22.60	[21.66; 23.56]
Harhay (2013)	1797	10958	16.40	[15.71; 17.11]
Harhay (2013)	1508	6472	23.30	[22.27; 24.35]
Supiyev (2015)	350	497	70.42	[66.20; 74.40]
**Random effects model**		**65229**	**31.50**	**[25.44; 37.89]**

Heterogeneity: I−squared=99.7% [99.6%; 99.7%], tau−squared=0.0558, p<0.0001

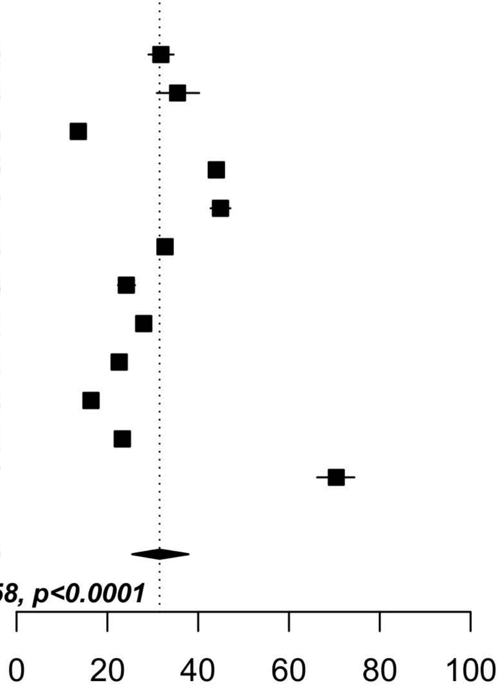

Author (year)	Hypertension cases	Sample size	Prevalence of hypertension	95% CI
Gupta (1994)	674	3148	21.41	[19.99; 22.89]
Gupta (1995)	688	2122	32.42	[30.43; 34.46]
Goel (1996)	113	1572	7.19	[5.96; 8.58]
Singh (1998)	822	3212	25.59	[24.09; 27.14]
Malhotra (1999)	114	2559	4.45	[3.69; 5.33]
Gurav (2001)	199	1432	13.90	[12.15; 15.80]
Gupta (2002)	415	1123	36.95	[34.12; 39.85]
Hazarika (2002)	617	1015	60.79	[57.71; 63.81]
Reddy (2002)	924	3307	27.94	[26.42; 29.50]
Swami (2002)	210	362	58.01	[52.74; 63.15]
Bharucha (2003)	879	2415	36.40	[34.48; 38.35]
Deepa (2003)	279	1262	22.11	[19.85; 24.50]
Hazarika (2003)	565	888	63.63	[60.36; 66.80]
Shanthirani (2003)	266	1262	21.08	[18.86; 23.43]
Gupta (2004)	42591	88653	48.04	[47.71; 48.37]
Hazarika (2004)	1059	3180	33.30	[31.66; 34.97]
Ahmad (2005)	1745	8432	20.69	[19.83; 21.58]
Das (2005)	401	1609	24.92	[22.82; 27.11]
Prabhakaran (2005)	637	2122	30.02	[28.07; 32.02]
Siddiqui (2005)	49	327	14.98	[11.30; 19.32]
Thankappan (2006)	1818	4955	36.69	[35.35; 38.05]
Chaturvedi (2007)	1039	2318	44.82	[42.78; 46.87]
Gupta (2007)	591	1127	52.44	[49.48; 55.39]
Mohan (2007)	470	2350	20.00	[18.40; 21.68]
Reddyet (2007)	6751	19975	33.80	[33.14; 34.46]
Vaidya (2007)	227	1000	22.70	[20.14; 25.42]
Wijewardene (2007)	1155	6047	19.10	[18.12; 20.11]
Agrawal (2008)	136	406	33.50	[28.92; 38.32]
Yadav (2008)	116	294	39.46	[33.83; 45.30]
Kusuma (2009)	83	453	18.32	[14.87; 22.20]
Midha (2009)	189	800	23.62	[20.72; 26.73]
Pednekar (2009)	132144	146827	90.00	[89.85; 90.15]
Pednekar (2009)	68498	148173	46.23	[45.97; 46.48]
Bhardwaj (2010)	392	1092	35.90	[33.05; 38.82]
Jonas (2010)	1041	4711	22.10	[20.92; 23.31]
Kar (2010)	143	400	35.75	[31.05; 40.66]
Kinra (2010)	401	1983	20.22	[18.47; 22.06]
Chataut (2011)	118	527	22.39	[18.90; 26.19]
Manimunda (2011)	492	975	50.46	[47.27; 53.65]
Norboo (2011)	1037	2800	37.04	[35.24; 38.86]
Thrift (2011)	169	1479	11.43	[9.85; 13.16]
Bansal (2012)	313	968	32.33	[29.39; 35.38]
Bharati (2012)	236	856	27.57	[24.60; 30.70]
Borah (2012)	407	916	44.43	[41.18; 47.72]
Chinnakali (2012)	85	211	40.28	[33.61; 47.24]
Dutta (2012)	293	1186	24.70	[22.27; 27.26]
Esam (2012)	138	504	27.38	[23.53; 31.50]
Gupta (2012)	1672	4073	41.05	[39.53; 42.58]
Gupta (2012)	248	739	33.56	[30.16; 37.09]
Jeemon (2012)	3564	10543	33.80	[32.90; 34.72]
Kaur (2012)	2247	10463	21.48	[20.69; 22.28]
Kaur (2012)	108	600	18.00	[15.01; 21.31]
Kokiwar (2012)	176	924	19.05	[16.56; 21.73]
Meshram (2012)	1671	4192	39.86	[38.38; 41.36]
Prasad (2012)	431	1178	36.59	[33.83; 39.41]
Prince (2012)	1133	1977	57.31	[55.09; 59.50]
Samuel (2012)	55	2218	2.48	[1.87; 3.22]
Vaidya (2012)	412	1218	33.83	[31.17; 36.56]
Vaidya (2012)	112	641	17.47	[14.61; 20.64]
Bhagyalaxmi (2013)	785	3489	22.50	[21.12; 23.92]
Gupta (2013)	2613	6106	42.79	[41.55; 44.05]
Khan (2013)	55	1679	3.28	[2.48; 4.24]
Adhikari (2014)	276	1240	22.26	[19.97; 24.68]
Lloyd−Sherlock (2014)	2338	7238	32.30	[31.22; 33.39]
Zaman (2014)	941	4073	23.10	[21.82; 24.43]
Bhansali (2015)	3698	14059	26.30	[25.58; 27.04]
Gupta (2015)	1959	6198	31.61	[30.45; 32.78]
Menon (2015)	5005	84456	5.93	[5.77; 6.09]
Rahman (2015)	1922	7876	24.40	[23.46; 25.37]
Ranasighe (2015)	1189	4482	26.53	[25.24; 27.85]
**Random effects model**		**662997**	**29.40**	**[22.34; 37.00]**

Heterogeneity: I−squared=100% [100%; 100%], tau−squared=0.4741, p<0.0001

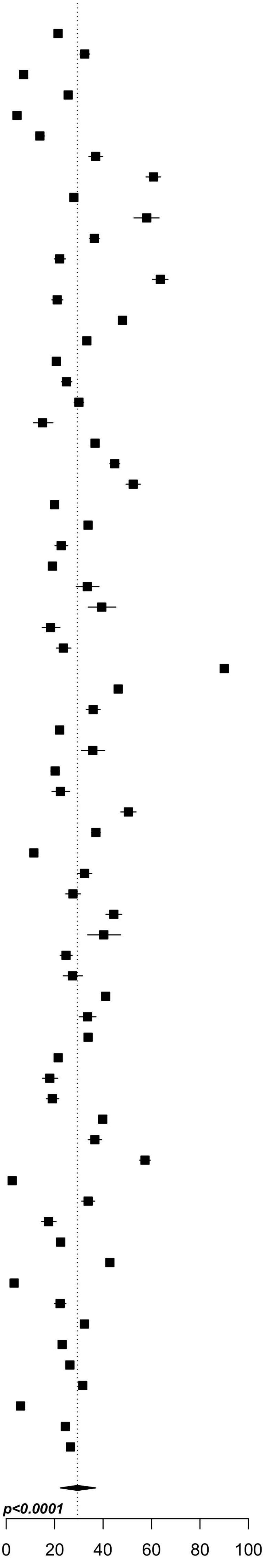

Author (year)	Hypertension cases	Sample size	Prevalence of hypertension	95% CI
Ordunez−Garcia (1998)	719	1633	44.03	[41.60; 46.48]
Barreto (2001)	574	2314	24.81	[23.06; 26.62]
Freitas (2001)	217	688	31.54	[28.08; 35.16]
Wu (2001)	1358	2272	59.77	[57.72; 61.80]
Gu (2002)	3287	13199	24.90	[24.17; 25.65]
Lorenzo (2002)	429	2282	18.80	[17.22; 20.46]
Matos (2003)	46	126	36.51	[28.12; 45.55]
Shapo (2003)	356	1120	31.79	[29.06; 34.60]
Gus (2004)	358	1063	33.68	[30.84; 36.61]
Lim (2004)	7059	21391	33.00	[32.37; 33.63]
Onal (2004)	150	423	35.46	[30.90; 40.23]
Ordunez (2005)	332	1667	19.92	[18.02; 21.92]
Almeida−Pititto (2006)	160	484	33.06	[28.88; 37.45]
Bahrami (2006)	2925	8999	32.50	[31.54; 33.48]
Lessa (2006)	430	1439	29.88	[27.53; 32.32]
Jardim (2007)	633	1739	36.40	[34.13; 38.71]
Medina−Lezama (2007)	295	1878	15.71	[14.09; 17.43]
Thorogood (2007)	171	402	42.54	[37.65; 47.53]
Capilheira (2008)	725	3100	23.39	[21.91; 24.92]
Erem (2008)	2116	4809	44.00	[42.59; 45.42]
Ordunez (2008)	316	1475	21.42	[19.35; 23.61]
Rampal (2008)	4570	16440	27.80	[27.11; 28.49]
Sparrenberger (2008)	600	1484	40.43	[37.92; 42.98]
Sun (2008)	17929	45390	39.50	[39.05; 39.95]
Azimi−Nezhad (2009)	1094	4519	24.21	[22.97; 25.49]
Grimsrud (2009)	727	4351	16.71	[15.61; 17.85]
Longo (2009)	681	2022	33.68	[31.62; 35.79]
Ramezani (2009)	778	3760	20.69	[19.41; 22.02]
Reichert (2009)	583	1696	34.38	[32.11; 36.69]
Rodrigues (2009)	702	1655	42.42	[40.02; 44.84]
Rosario (2009)	302	1003	30.11	[27.28; 33.05]
Zhang (2009)	2008	4141	48.49	[46.96; 50.03]
Cipullo (2010)	762	1717	44.38	[42.01; 46.77]
Dorobantu (2010)	906	2017	44.92	[42.73; 47.12]
Ebrahimi (2010)	5215	29971	17.40	[16.97; 17.83]
Lee (2010)	9852	39252	25.10	[24.67; 25.53]
Nascente (2010)	382	1168	32.71	[30.02; 35.48]
Swaddiwudhipong (2010)	1571	5273	29.79	[28.56; 31.05]
Wu (2010)	869	2074	41.90	[39.76; 44.06]
Altun (2012)	3515	10748	32.70	[31.82; 33.60]
Dogan (2012)	492	2035	24.18	[22.33; 26.10]
Hendriks (2012)	555	1733	32.03	[29.83; 34.28]
Hofelmann (2012)	688	1720	40.00	[37.67; 42.36]
Kerkhoff (2012)	635	1858	34.18	[32.02; 36.38]
Lyra (2012)	127	198	64.14	[57.04; 70.82]
Prince (2012)	2176	2944	73.91	[72.29; 75.49]
Prince (2012)	1538	2000	76.90	[74.99; 78.73]
Prince (2012)	1422	1789	79.49	[77.54; 81.34]
Prince (2012)	1303	2157	60.41	[58.31; 62.48]
Prince (2012)	1259	2001	62.92	[60.76; 65.04]
Prince (2012)	961	1929	49.82	[47.56; 52.07]
Harhay (2013)	1797	10958	16.40	[15.71; 17.11]
Harhay (2013)	1508	6472	23.30	[22.27; 24.35]
Kandala (2013)	4133	13596	30.40	[29.63; 31.18]
Kiau (2013)	3650	4933	73.99	[72.74; 75.21]
Mendes (2013)	395	842	46.91	[43.50; 50.35]
Peer (2013)	460	1099	41.86	[38.92; 44.84]
Peltzer (2013)	2968	3840	77.29	[75.93; 78.61]
Selem (2013)	232	535	43.36	[39.12; 47.68]
Silva (2013)	695	1720	40.41	[38.08; 42.77]
Veghari (2013)	741	3497	21.19	[19.85; 22.58]
Amiri (2014)	431	1096	39.32	[36.42; 42.29]
Fan (2014)	4673	18772	24.89	[24.28; 25.52]
Feng (2014)	5295	13707	38.63	[37.81; 39.45]
Lloyd−Sherlock (2014)	7942	13348	59.50	[58.66; 60.33]
Lloyd−Sherlock (2014)	1328	2281	58.22	[56.16; 60.25]
Lloyd−Sherlock (2014)	2976	3820	77.91	[76.56; 79.21]
Posso (2014)	1008	3406	29.59	[28.07; 31.16]
Wang (2014)	11905	37141	32.05	[31.58; 32.53]
Zhao (2014)	3086	6324	48.80	[47.56; 50.04]
Almeida (2015)	750	1410	53.19	[50.55; 55.82]
Bernabe Ortiz (2015)	518	3218	16.10	[14.84; 17.41]
Botha (2015)	191	429	44.52	[39.75; 49.37]
Bresan (2015)	164	355	46.20	[40.92; 51.54]
Chen (2015)	850	2208	38.50	[36.46; 40.56]
Gu (2015)	2922	7137	40.94	[39.80; 42.09]
Guo (2015)	6981	24410	28.60	[28.03; 29.17]
Hou (2015)	1754	3797	46.19	[44.60; 47.80]
Iazdanpanah (2015)	72	944	7.63	[6.02; 9.51]
Ke (2015)	479	1410	33.97	[31.50; 36.51]
Khalifeh (2015)	224	672	33.33	[29.77; 37.04]
Li (2015)	545	2026	26.90	[24.98; 28.89]
Lu (2015)	1477	4675	31.59	[30.26; 32.95]
Matar (2015)	626	1697	36.89	[34.59; 39.23]
Sepanlou (2015)	18694	46674	40.05	[39.61; 40.50]
Supiyev (2015)	350	497	70.42	[66.20; 74.40]
Unger (2015)	1162	5649	20.57	[19.52; 21.65]
Vieira (2015)	111	491	22.61	[18.98; 26.57]
Wang (2015)	6372	15172	42.00	[41.21; 42.79]
Wei (2015)	1549	3778	41.00	[39.43; 42.59]
**Random effects model**		**545584**	**37.79**	**[35.01; 40.61]**

Heterogeneity: I−squared=99.8% [99.8%; 99.8%], tau−squared=0.0773, p<0.0001

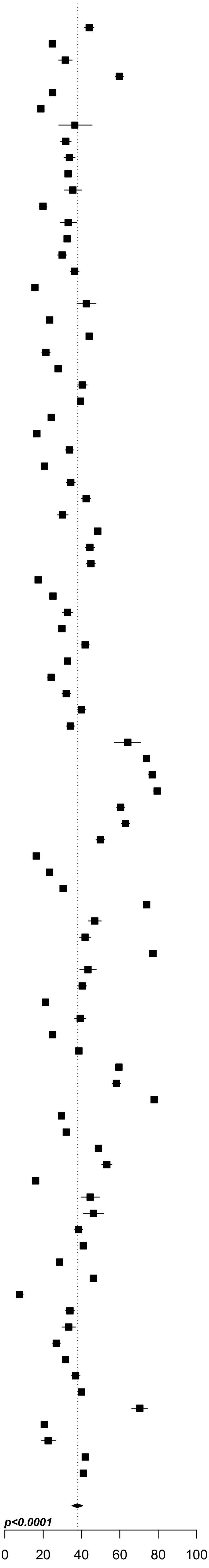

Author (year)	Hypertension cases	Sample size	Prevalence of hypertension	95% CI
Gupta (1994)	674	3148	21.41	[19.99; 22.89]
Gupta (1995)	688	2122	32.42	[30.43; 34.46]
Ibrahim (1995)	1771	6733	26.30	[25.25; 27.37]
Goel (1996)	113	1572	7.19	[5.96; 8.58]
Kaufman (1996)	144	598	24.08	[20.70; 27.71]
Singh (1998)	822	3212	25.59	[24.09; 27.14]
Malhotra (1999)	114	2559	4.45	[3.69; 5.33]
Gurav (2001)	199	1432	13.90	[12.15; 15.80]
Shmulewitz (2001)	372	2188	17.00	[15.45; 18.64]
Gupta (2002)	415	1123	36.95	[34.12; 39.85]
Hazarika (2002)	617	1015	60.79	[57.71; 63.81]
Reddy (2002)	924	3307	27.94	[26.42; 29.50]
Swami (2002)	210	362	58.01	[52.74; 63.15]
Amoah (2003)	1339	4733	28.29	[27.01; 29.60]
Bharucha (2003)	879	2415	36.40	[34.48; 38.35]
Deepa (2003)	279	1262	22.11	[19.85; 24.50]
Hazarika (2003)	565	888	63.63	[60.36; 66.80]
Shanthirani (2003)	266	1262	21.08	[18.86; 23.43]
Cappuccio (2004)	291	1013	28.73	[25.96; 31.62]
Gupta (2004)	42591	88653	48.04	[47.71; 48.37]
Hazarika (2004)	1059	3180	33.30	[31.66; 34.97]
Ahmad (2005)	1745	8432	20.69	[19.83; 21.58]
Das (2005)	401	1609	24.92	[22.82; 27.11]
Erhun (2005)	210	1000	21.00	[18.51; 23.66]
Prabhakaran (2005)	637	2122	30.02	[28.07; 32.02]
Siddiqui (2005)	49	327	14.98	[11.30; 19.32]
Agyemang (2006)	421	1431	29.42	[27.07; 31.86]
Kamadjeu (2006)	2413	10011	24.10	[23.27; 24.95]
Minh (2006)	281	1996	14.08	[12.58; 15.68]
Mishra (2006)	1060	7796	13.60	[12.84; 14.38]
Thankappan (2006)	1818	4955	36.69	[35.35; 38.05]
Chaturvedi (2007)	1039	2318	44.82	[42.78; 46.87]
Duda (2007)	721	1321	54.58	[51.85; 57.29]
Gupta (2007)	591	1127	52.44	[49.48; 55.39]
Kengme (2007)	532	2559	20.79	[19.23; 22.41]
Mohan (2007)	470	2350	20.00	[18.40; 21.68]
Omuemu (2007)	119	590	20.17	[17.00; 23.64]
Reddyet (2007)	6751	19975	33.80	[33.14; 34.46]
Wijewardene (2007)	1155	6047	19.10	[18.12; 20.11]
Agrawal (2008)	136	406	33.50	[28.92; 38.32]
Yadav (2008)	116	294	39.46	[33.83; 45.30]
Diaz (2009)	11907	19519	61.00	[60.31; 61.69]
Kusuma (2009)	83	453	18.32	[14.87; 22.20]
Midha (2009)	189	800	23.62	[20.72; 26.73]
Pednekar (2009)	132144	146827	90.00	[89.85; 90.15]
Pednekar (2009)	68498	148173	46.23	[45.97; 46.48]
Bhardwaj (2010)	392	1092	35.90	[33.05; 38.82]
Ekwunife (2010)	160	756	21.16	[18.30; 24.25]
Jonas (2010)	1041	4711	22.10	[20.92; 23.31]
Kar (2010)	143	400	35.75	[31.05; 40.66]
Kinra (2010)	401	1983	20.22	[18.47; 22.06]
Oladapo (2010)	415	2000	20.75	[18.99; 22.59]
Sani (2010)	77	300	25.67	[20.82; 31.00]
Thuy (2010)	320	910	35.16	[32.06; 38.37]
Ulasi (2010)	478	1458	32.78	[30.38; 35.26]
Manimunda (2011)	492	975	50.46	[47.27; 53.65]
Norboo (2011)	1037	2800	37.04	[35.24; 38.86]
Thrift (2011)	169	1479	11.43	[9.85; 13.16]
Ulasi (2011)	290	688	42.15	[38.43; 45.94]
Wokoma (2011)	42	152	27.63	[20.70; 35.46]
Bansal (2012)	313	968	32.33	[29.39; 35.38]
Berraho (2012)	370	525	70.48	[66.37; 74.35]
Bharati (2012)	236	856	27.57	[24.60; 30.70]
Borah (2012)	407	916	44.43	[41.18; 47.72]
Chinnakali (2012)	85	211	40.28	[33.61; 47.24]
Dutta (2012)	293	1186	24.70	[22.27; 27.26]
Dzudie (2012)	1007	2120	47.50	[45.36; 49.65]
Esam (2012)	138	504	27.38	[23.53; 31.50]
Gupta (2012)	248	739	33.56	[30.16; 37.09]
Gupta (2012)	1672	4073	41.05	[39.53; 42.58]
Hendriks (2012)	562	2678	20.99	[19.46; 22.58]
Jeemon (2012)	3564	10543	33.80	[32.90; 34.72]
Kaur (2012)	2247	10463	21.48	[20.69; 22.28]
Kaur (2012)	108	600	18.00	[15.01; 21.31]
Kokiwar (2012)	176	924	19.05	[16.56; 21.73]
Macia (2012)	327	500	65.40	[61.05; 69.57]
Meshram (2012)	1671	4192	39.86	[38.38; 41.36]
Oladimeji (2012)	232	801	28.96	[25.84; 32.24]
Prasad (2012)	431	1178	36.59	[33.83; 39.41]
Prince (2012)	1133	1977	57.31	[55.09; 59.50]
Samuel (2012)	55	2218	2.48	[1.87; 3.22]
Adebayo (2013)	264	1000	26.40	[23.69; 29.25]
Asekun−Olarinmoye (2013)	34	259	13.13	[9.27; 17.86]
Bhagyalaxmi (2013)	785	3489	22.50	[21.12; 23.92]
Ekanem (2013)	208	442	47.06	[42.33; 51.83]
Gupta (2013)	2613	6106	42.79	[41.55; 44.05]
Ha (2013)	552	2368	23.31	[21.62; 25.07]
Harhay (2013)	1707	7552	22.60	[21.66; 23.56]
Harhay (2013)	3025	10802	28.00	[27.16; 28.86]
Modesti (2013)	789	10242	7.70	[7.19; 8.24]
Ogah (2013)	1566	2928	53.48	[51.66; 55.30]
Ogunmola (2013)	69	104	66.35	[56.42; 75.32]
Okpechi (2013)	936	2983	31.38	[29.71; 33.08]
Pessinaba (2013)	655	1424	46.00	[43.38; 48.63]
Awosan (2014)	114	390	29.23	[24.76; 34.02]
Awuah (2014)	202	714	28.29	[25.01; 31.75]
Duboz (2014)	165	600	27.50	[23.96; 31.26]
Lloyd−Sherlock (2014)	2338	7238	32.30	[31.22; 33.39]
Lloyd−Sherlock (2014)	2692	4716	57.08	[55.66; 58.50]
Nguyen (2014)	442	3779	11.70	[10.69; 12.76]
Oluyombo (2014)	354	750	47.20	[43.58; 50.84]
Bhansali (2015)	3698	14059	26.30	[25.58; 27.04]
Bushara (2015)	420	1099	38.22	[35.33; 41.16]
De Souza (2015)	432	1729	24.99	[22.96; 27.10]
Do (2015)	3560	17199	20.70	[20.10; 21.31]
Ezeala−Adekaibe (2015)	406	774	52.45	[48.87; 56.02]
Gupta (2015)	1959	6198	31.61	[30.45; 32.78]
Ibekwe (2015)	57	272	20.96	[16.28; 26.28]
Isara (2015)	319	845	37.75	[34.47; 41.12]
Kingue (2015)	4595	15470	29.70	[28.98; 30.43]
Lim (2015)	22060	58985	37.40	[37.01; 37.79]
Menon (2015)	5005	84456	5.93	[5.77; 6.09]
Minicuci (2015)	2413	4724	51.08	[49.64; 52.51]
Oguoma (2015)	151	422	35.78	[31.20; 40.56]
Ranasighe (2015)	1189	4482	26.53	[25.24; 27.85]
Seck (2015)	147	627	23.44	[20.18; 26.96]
Sowemimo (2015)	267	806	33.13	[29.88; 36.50]
Ugwuja (2015)	62	267	23.22	[18.29; 28.76]
**Random effects model**		**873391**	**31.14**	**[26.11; 36.41]**

Heterogeneity: I−squared=100% [100%; 100%], tau−squared=0.3789, p<0.0001

Author (year)	Hypertension cases	Sample size	Prevalence of hypertension	95% CI
Edwards (2000)	520	1698	30.62	[28.44; 32.88]
Jean−Baptiste (2006)	524	1113	47.08	[44.11; 50.06]
Niakara (2007)	822	2044	40.22	[38.08; 42.38]
Vaidya (2007)	227	1000	22.70	[20.14; 25.42]
Damasceno (2009)	1100	3323	33.10	[31.50; 34.73]
Tesfaye (2009)	1114	3713	30.00	[28.53; 31.51]
Wamala (2009)	256	842	30.40	[27.31; 33.63]
Chataut (2011)	118	527	22.39	[18.90; 26.19]
Maher (2011)	1469	6678	22.00	[21.01; 23.01]
Awoke (2012)	192	679	28.28	[24.92; 31.83]
Hendriks (2012)	426	2111	20.18	[18.49; 21.96]
Hendriks (2012)	199	1046	19.02	[16.69; 21.54]
Mayega (2012)	339	1656	20.47	[18.55; 22.50]
Msyamboza (2012)	1237	3727	33.19	[31.68; 34.73]
Vaidya (2012)	412	1218	33.83	[31.17; 36.56]
Vaidya (2012)	112	641	17.47	[14.61; 20.64]
Khan (2013)	55	1679	3.28	[2.48; 4.24]
Musinguzi (2013)	995	4563	21.81	[20.62; 23.03]
Adhikari (2014)	276	1240	22.26	[19.97; 24.68]
Doulougou (2014)	380	2041	18.62	[16.95; 20.38]
Doulougou (2014)	139	1481	9.39	[7.95; 10.99]
Helelo (2014)	116	518	22.39	[18.87; 26.23]
Moges (2014)	9	68	13.24	[6.23; 23.64]
Zaman (2014)	941	4073	23.10	[21.82; 24.43]
Abebe (2015)	614	2200	27.91	[26.04; 29.83]
Akpan (2015)	595	1565	38.02	[35.61; 40.48]
Angaw (2015)	179	655	27.33	[23.95; 30.91]
Anteneh (2015)	170	678	25.07	[21.85; 28.51]
Asilu (2015)	950	7741	12.27	[11.55; 13.02]
Muusinguzi (2015)	895	4432	20.19	[19.02; 21.41]
Rahman (2015)	1922	7876	24.40	[23.46; 25.37]
Sander (2015)	34	426	7.98	[5.59; 10.97]
Wandera (2015)	376	2382	15.79	[14.34; 17.31]
**Random effects model**		**75634**	**23.11**	**[20.13; 26.23]**

Heterogeneity: I−squared=99% [98.9%; 99.1%], tau−squared=0.0438, p<0.0001

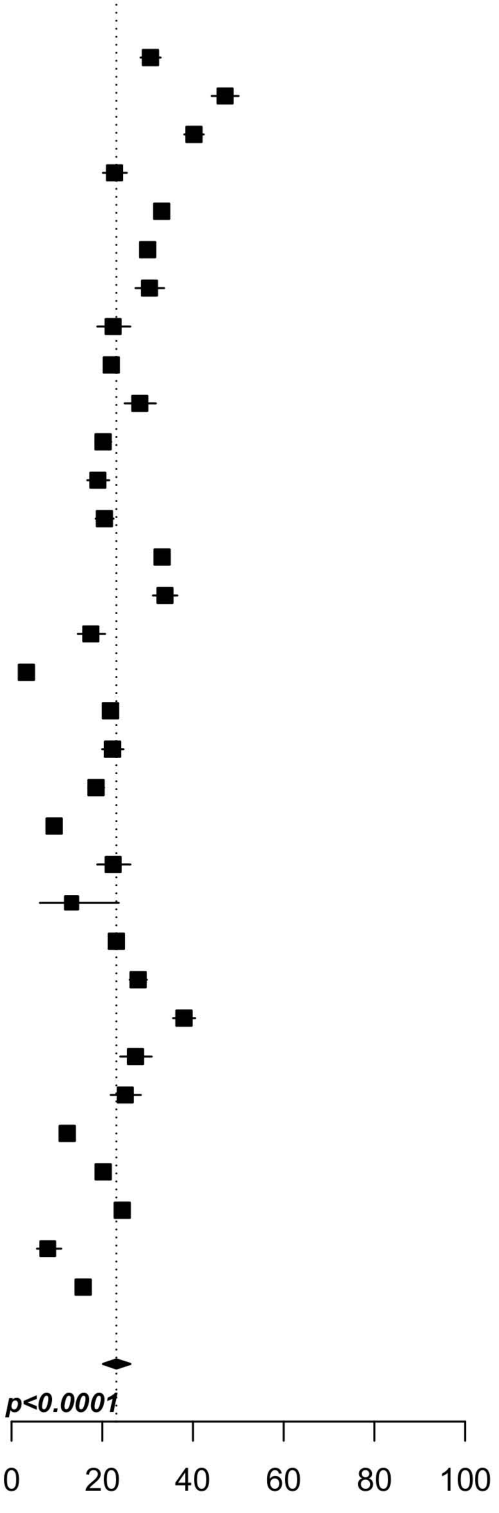

Author (year)	Hypertension cases	Sample size	Prevalence of hypertension	95% CI
Gupta (1995)	688	2122	32.42	[30.43; 34.46]
Ordunez−Garcia (1998)	719	1633	44.03	[41.60; 46.48]
Singh (1998)	822	3212	25.59	[24.09; 27.14]
Freitas (2001)	217	688	31.54	[28.08; 35.16]
Gurav (2001)	199	1432	13.90	[12.15; 15.80]
Gupta (2002)	415	1123	36.95	[34.12; 39.85]
Hazarika (2002)	617	1015	60.79	[57.71; 63.81]
Lorenzo (2002)	429	2282	18.80	[17.22; 20.46]
Reddy (2002)	924	3307	27.94	[26.42; 29.50]
Amoah (2003)	1339	4733	28.29	[27.01; 29.60]
Deepa (2003)	279	1262	22.11	[19.85; 24.50]
Shanthirani (2003)	266	1262	21.08	[18.86; 23.43]
Shapo (2003)	356	1120	31.79	[29.06; 34.60]
Gupta (2004)	42591	88653	48.04	[47.71; 48.37]
Onal (2004)	150	423	35.46	[30.90; 40.23]
Das (2005)	401	1609	24.92	[22.82; 27.11]
Erhun (2005)	210	1000	21.00	[18.51; 23.66]
Ordunez (2005)	332	1667	19.92	[18.02; 21.92]
Prabhakaran (2005)	637	2122	30.02	[28.07; 32.02]
Siddiqui (2005)	49	327	14.98	[11.30; 19.32]
Almeida−Pititto (2006)	160	484	33.06	[28.88; 37.45]
Jean−Baptiste (2006)	524	1113	47.08	[44.11; 50.06]
Chaturvedi (2007)	1039	2318	44.82	[42.78; 46.87]
Duda (2007)	721	1321	54.58	[51.85; 57.29]
Gupta (2007)	591	1127	52.44	[49.48; 55.39]
Jardim (2007)	633	1739	36.40	[34.13; 38.71]
Kengme (2007)	532	2559	20.79	[19.23; 22.41]
Medina−Lezama (2007)	295	1878	15.71	[14.09; 17.43]
Niakara (2007)	822	2044	40.22	[38.08; 42.38]
Capilheira (2008)	725	3100	23.39	[21.91; 24.92]
Sparrenberger (2008)	600	1484	40.43	[37.92; 42.98]
Sun (2008)	17929	45390	39.50	[39.05; 39.95]
Yadav (2008)	116	294	39.46	[33.83; 45.30]
Kusuma (2009)	83	453	18.32	[14.87; 22.20]
Longo (2009)	681	2022	33.68	[31.62; 35.79]
Reichert (2009)	583	1696	34.38	[32.11; 36.69]
Rodrigues (2009)	702	1655	42.42	[40.02; 44.84]
Tesfaye (2009)	1114	3713	30.00	[28.53; 31.51]
Zhang (2009)	2008	4141	48.49	[46.96; 50.03]
Cipullo (2010)	762	1717	44.38	[42.01; 46.77]
Ekwunife (2010)	160	756	21.16	[18.30; 24.25]
Jonas (2010)	1041	4711	22.10	[20.92; 23.31]
Lee (2010)	9852	39252	25.10	[24.67; 25.53]
Sani (2010)	77	300	25.67	[20.82; 31.00]
Thuy (2010)	320	910	35.16	[32.06; 38.37]
Ulasi (2011)	290	688	42.15	[38.43; 45.94]
Awoke (2012)	192	679	28.28	[24.92; 31.83]
Dzudie (2012)	1007	2120	47.50	[45.36; 49.65]
Gupta (2012)	248	739	33.56	[30.16; 37.09]
Hendriks (2012)	199	1046	19.02	[16.69; 21.54]
Hendriks (2012)	555	1733	32.03	[29.83; 34.28]
Hofelmann (2012)	688	1720	40.00	[37.67; 42.36]
Kerkhoff (2012)	635	1858	34.18	[32.02; 36.38]
Lyra (2012)	127	198	64.14	[57.04; 70.82]
Oladimeji (2012)	232	801	28.96	[25.84; 32.24]
Prasad (2012)	431	1178	36.59	[33.83; 39.41]
Ekanem (2013)	208	442	47.06	[42.33; 51.83]
Mendes (2013)	395	842	46.91	[43.50; 50.35]
Peer (2013)	460	1099	41.86	[38.92; 44.84]
Pessinaba (2013)	655	1424	46.00	[43.38; 48.63]
Selem (2013)	232	535	43.36	[39.12; 47.68]
Silva (2013)	695	1720	40.41	[38.08; 42.77]
Veghari (2013)	741	3497	21.19	[19.85; 22.58]
Amiri (2014)	431	1096	39.32	[36.42; 42.29]
Awosan (2014)	114	390	29.23	[24.76; 34.02]
Awuah (2014)	202	714	28.29	[25.01; 31.75]
Doulougou (2014)	380	2041	18.62	[16.95; 20.38]
Duboz (2014)	165	600	27.50	[23.96; 31.26]
Moges (2014)	9	68	13.24	[6.23; 23.64]
Mooes (2014)	9	68	13.24	[6.23; 23.64]
Wang (2014)	11905	37141	32.05	[31.58; 32.53]
Ezeala−Adekaibe (2015)	406	774	52.45	[48.87; 56.02]
Gupta (2015)	1959	6198	31.61	[30.45; 32.78]
Ke (2015)	479	1410	33.97	[31.50; 36.51]
Kingue (2015)	4595	15470	29.70	[28.98; 30.43]
Li (2015)	545	2026	26.90	[24.98; 28.89]
Lu (2015)	1477	4675	31.59	[30.26; 32.95]
Unger (2015)	1162	5649	20.57	[19.52; 21.65]
Vieira (2015)	111	491	22.61	[18.98; 26.57]
Wei (2015)	1549	3778	41.00	[39.43; 42.59]
**Random effects model**		**352077**	**32.65**	**[30.37; 34.97]**

Heterogeneity: I−squared=99.5% [99.5%; 99.5%], tau−squared=0.0487, p<0.0001

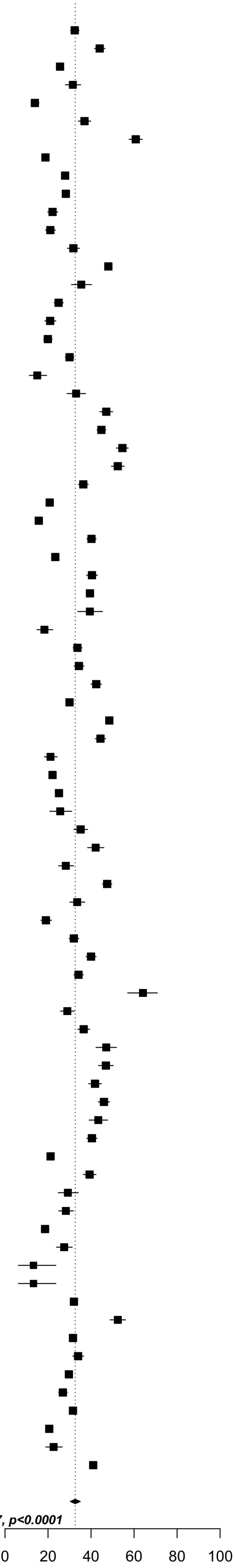

Supplement: Supplemental Digital Content [file medi-94-e1959-s001.pdf]
